# Trait biases in microbial reference genomes

**DOI:** 10.1038/s41597-023-01994-7

**Published:** 2023-02-09

**Authors:** Sage Albright, Stilianos Louca

**Affiliations:** 1 Department of Biology, University of Oregon, Eugene, USA; 2 Institute of Ecology and Evolution, University of Oregon, Eugene, USA

**Keywords:** Biodiversity, Microbial ecology, Microbiology techniques

## Abstract

Common culturing techniques and priorities bias our discovery towards specific traits that may not be representative of microbial diversity in nature. So far, these biases have not been systematically examined. To address this gap, here we use 116,884 publicly available metagenome-assembled genomes (MAGs, completeness ≥80%) from 203 surveys worldwide as a culture-independent sample of bacterial and archaeal diversity, and compare these MAGs to the popular RefSeq genome database, which heavily relies on cultures. We compare the distribution of 12,454 KEGG gene orthologs (used as trait proxies) in the MAGs and RefSeq genomes, while controlling for environment type (ocean, soil, lake, bioreactor, human, and other animals). Using statistical modeling, we then determine the conditional probabilities that a species is represented in RefSeq depending on its genetic repertoire. We find that the majority of examined genes are significantly biased for or against in RefSeq. Our systematic estimates of gene prevalences across bacteria and archaea in nature and gene-specific biases in reference genomes constitutes a resource for addressing these issues in the future.

## Introduction

Culturing remains the golden standard for studying bacterial and archaeal (henceforth “prokaryotic” for brevity) physiology, metabolism and pathogenicity^1^, and for obtaining high-quality genome sequences, such as those in the NCBI RefSeq reference sequence database^2^. The thousands of prokaryotic genomes now available in RefSeq, in turn, enable large-scale analyses that yield insight into the processes shaping microbial genome structure and evolution^3–9^, and also form the starting pool for curated gene ontologies or databases such as eggNOG^10^ and rrnDB^11^. Reference genome databases and culture-based phenotype databases also enable predictions of the likely gene contents and traits of other less studied prokaryotic clades seen in environmental samples based on phylogenetic relationships, e.g., using tools such as PICRUSt^12^, Tax4Fun^13^ and FAPROTAX^14^. However, to date the vast majority of extant prokaryotic diversity remains uncultured and lacking a whole genome sequence, partly due to the difficulties associated with determining the proper growth conditions for each species. Conventional culturing techniques, the fact that pure cultures must grow in the absence of syntrophic partners, and typical research priorities are thought to bias our discovery towards traits that may not be representative of the broader prokaryotic diversity in nature^1,15^. These biases can distort our vision of prokaryotic diversity, limit our capacity to discover new useful biochemical functions^15^, introduce biases in comparative phylogenetic and other evolutionary analyses^16^, and most likely bias phylogeny-based predictions of gene content and traits in uncultured organisms^16^. For example, trait biases in RefSeq are expected to cause corresponding prediction biases in PICRUSt and Tax4Fun. Systematically quantifying the true distribution of traits across prokaryotic clades and determining trait biases in reference genome databases (and by extension, in prokaryotic cultures) is required for assessing the extent of these important issues and addressing them in the future. To date such an analysis across a broad range of clades and environments is lacking, one reason being that it was until recently impossible to efficiently recover a large culture-independent set of microbial genomes from natural environments.

Recent advances in genome-resolved metagenomics now enable the recovery of nearly-complete prokaryotic genomes from complex natural microbial communities without the need for culturing^17–20^. Here we use 116,884 previously published prokaryotic metagenome-assembled genomes (MAGs) from around the world to obtain a culture-independent sample of extant prokaryotic diversity in nature. We use this collection of MAGs to estimate the true prevalences of thousands of different genes (considered here as proxies of traits) across prokaryotic clades. We compare these gene prevalences to those in the widely used NCBI RefSeq prokaryotic reference genome database^2^, and quantify gene-dependent biases of clades represented in RefSeq. The RefSeq genome database was chosen for comparison as it is one of the oldest, most comprehensive and most widely used reference genome databases, and because it is largely based on cultured organisms, although we acknowledge that some cultured organisms have not yet had their genome sequenced and accessioned in RefSeq. We use statistical models to examine to what extent specific genes influence the probability of a random MAG being represented in RefSeq to at least 95% average nucleotide identity (ANI), which is a common modern measure for delineating prokaryotic species^6,21–24^. To account for obvious environmental preferences in both culturing and metagenomic sequencing efforts, we perform our analyses separately for different environment types, including the human microbiome, the microbiomes of non-human animals (henceforth “animals” for brevity), bioreactors, the ocean, soil, and lakes.

## Results

### A diverse collection of MAGs

Our collection of 116,884 prokaryotic MAGs was obtained from 203 distinct studies, covering the human microbiome (20 studies), other animals (30), bioreactors (including wastewater treatment plants, 35), the ocean (including estuaries and coastal lagoons, 72), soils (23) and lakes (28) (overview in Supplemental Table S2), and covers over 150 different phyla (Supplemental Fig. S2). All MAGs were estimated to be at least 80% complete and exhibit no more than 5% contamination, based on a set of universal single-copy marker genes (details in Methods section, overview in Supplemental Fig. S1). To avoid redundancies in species representation, we clustered MAGs into species genome bins (SGBs) based on an average nucleotide identity (ANI) threshold of 95%^6,21–24^. The probability that a randomly chosen prokaryotic species is represented in RefSeq (henceforth “coverage”) was determined based on the fraction of MAG-SGB representatives that could be matched to at least one RefSeq genome at ANI ≥95%.

Overall, we found that only a small fraction of MAG-SGBs could be matched to a RefSeq genome, although strong differences existed between environments. Notably, by far the highest coverage was found for human-associated prokaryotes (33%) and the lowest coverages were found for lakes (2.2%) and soil (4.9%) (overview in Supplemental Table S2). This is consistent with a previous study that showed that the cultured fraction of prokaryotes associated with the human gut is substantially above the average across environments^25^, and confirms the general expectation that only a small fraction of non-human-associated prokaryotic clades has been cultured. Our coverage estimates are also comparable to the global coverage estimated previously by Zhang *et al*.^26^ based on 16S SSU rRNA amplicon sequences (~2.1%).

### Gene prevalence estimates

To estimate how the prevalence of various genes differs between MAGs and prokaryotic RefSeq genomes, we searched for KEGG gene orthologs (KO’s) in MAGs as well as in RefSeq genomes using Hidden Markov Models (HMMs) from the KOfam database^27^ (annotation summaries in Supplemental Table S1). We chose to focus on KEGG because (a) it is widely used in microbial ecology, (b) it provides ready-to-use HMMs for more accurate gene annotation than BLAST-based searches, and (c) its functional focus facilitates the interpretation of the gene-specific biases examined here. In order to avoid Eukaryote-specific genes, only genes found in at least one MAG or prokaryotic RefSeq genome were considered (12,454 genes). To eliminate redundancies in species representation in RefSeq, we clustered RefSeq genomes into species bins based on their provided species-level taxon-IDs (STIBs), and only considered a single representative per STIB. Note that we focus on the true prevalence of each gene in the original populations represented by the MAG-SGBs or RefSeq STIBs (henceforth denoted *α*), and not merely the detection rate of that gene in the MAG-SGBs or RefSeq STIBs; indeed, mere detection rates are generally lower than true prevalences due to the incompleteness of many MAGs and some RefSeq genomes. To account for the incompleteness of each MAG and RefSeq genome in our gene prevalence estimates, we used an appropriate probabilistic model that we fitted via maximum-likelihood (details in Methods).

Overall, we found that gene detection rates were strongly skewed towards the lower end regardless of environment, with the majority of genes detected in fewer than 5% of MAG-SGBs and RefSeq STIBs (histograms in Supplemental Fig. S3 and Supplemental Fig. S4). As expected, estimated gene prevalences in MAG-SGBs (i.e., accounting for MAG incompleteness) were generally greater than mere gene detection rates (Supplemental Fig. S6), although gene prevalences still exhibited a strong skew towards lower values regardless of environment (Supplemental Fig. S5). MAG-SGB-based gene prevalences exhibited substantial and clearly significant positive correlations between environments, i.e., a gene that was widespread in species from one environment also tended to be widespread in species from other environments (Pearson *r* ≥ 0.840 and *P* < 0.001 for all environment pairs, Supplemental Fig. S7). That said, the degree to which gene prevalences correlated between environments varied considerably. For example, the strongest correlation was found between lakes and bioreactors (*r* = 0.991), between humans and other animals (*r* = 0.990) and between lakes and ocean (*r* = 0.985), while the weakest correlations were found between soil and humans (*r* = 0.840) and between soil and other animals (*r* = 0.846). These results suggest that the selective forces determining the prevalences of various genes across species tend to be somewhat similar across environments, and tend to be particularly similar between the human microbiome are other animal microbiomes, and between lakes, bioreactors and the ocean.

**Fig. 1:**
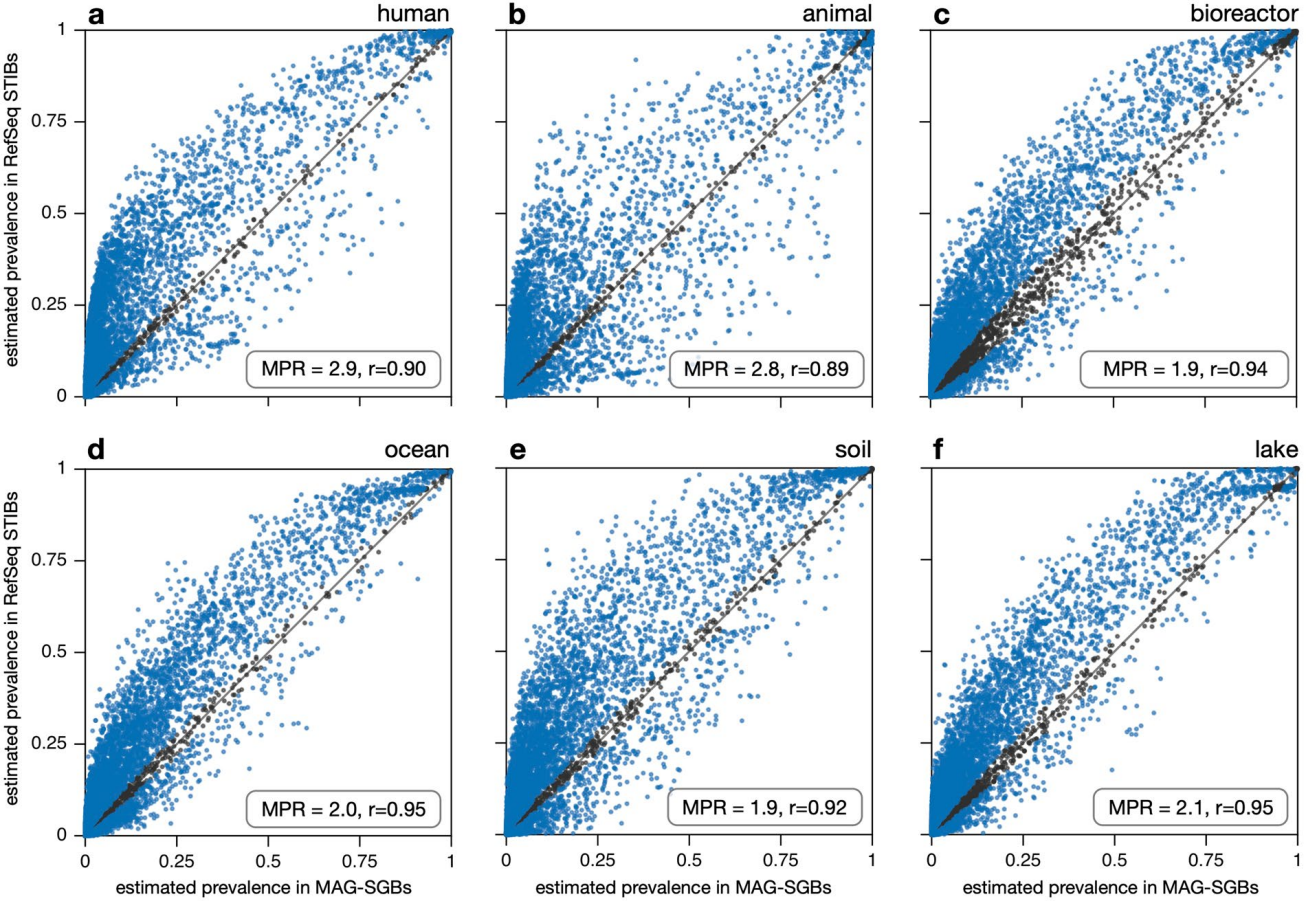
Gene prevalences (MAG-SGBs vs RefSeq-STIBs). Estimated gene prevalences in MAG-SGBs (horizontal axes) compared to RefSeq STIBs (vertical axes), separately for SGBs/STIBs associated with (**a**) humans, (**b**) other animals, (**c**) bioreactors, (**d**) ocean, (**e**) soil and (**f**) lakes. Every dot represents a distinct gene (KEGG Ortholog). Gene prevalences refer to the populations represented by the MAGs and STIBs, i.e., correcting for genome incompleteness. Blue dots denote genes whose estimated prevalence is significantly different in SGBs compared to STIBs (i.e., the 95% confidence intervals of the two estimates do not overlap), while black dots denote genes whose prevalence is not significantly different. The diagonal is shown for reference. The median prevalence ratio (MPR, prevalence in STIBs divided by the prevalence in SGBs, median taken across genes) and the Pearson correlation coefficient (*r*) are shown in each plot; all correlations were highly significant based on a permutation test (P < 0.001). For similar plots showing genome-size adjusted gene prevalences in STIBs see Supplemental Fig. S8. Abbreviations: MAG, metagenome-assembled genome; SGB, species genome bin; STIB, species taxon-ID bin; ANI, average nucleotide identity.

**Fig. 2:**
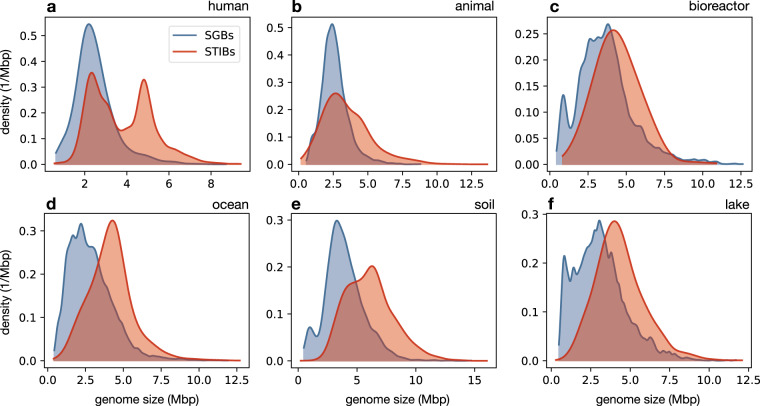
Genome sizes (MAG-SGBs vs RefSeq-STIBs). Kernel density estimates (KDE) of the genome size distribution among MAG-SGBs (blue curves, correcting for incompleteness) and RefSeq STIBs (red curves), separately for each environment. In all cases, the mean and median genome size among MAG-SGBs was substantially lower than among RefSeq STIBs. For mean genome sizes and KDE bandwidths see Supplemental Table S3.

Estimated gene prevalences in MAG-SGBs correlated positively with those in RefSeq STIBs, with Pearson correlation coefficients (*r*) ranging between 0.89 and 0.95 depending on the environment (*P* < 0.001 in all cases, Fig. 1). While these correlations may seem high, we point out that they are generally similar to the correlations observed between environments (discussed in the previous paragraph), in other words differences between MAG-SGBs and RefSeq STIBs (when controlling for environment) are comparable to differences between environments. This compromises the ecological conclusions that one may draw based on gene prevalences in reference databases such as RefSeq. In fact, the vast majority of genes displayed significantly different prevalences between MAG-SGBs and RefSeq STIBs, with a general tendency for gene prevalences to be higher among RefSeq STIBs than among MAG-SGBs. Specifically, the median ratio between MAG-SGB-based prevalences and RefSeq STIB-based prevalences (“median prevalence ratio”, or MPR) was substantially above 1 in all environments, ranging from 1.9 for bioreactors and soil up to 2.9 for humans and 2.8 for non-human animals. This tendency of genes to be more prevalent in RefSeq STIBs than MAG-SGBs could be caused by three distinct but not mutually exclusive mechanisms: First, there may be a general bias in RefSeq towards larger genomes. Indeed, prokaryotes with larger genomes tend to be more metabolically versatile and thus likely less dependent on syntrophic partners, which facilitates their culturing^28–30^. Consistent with this interpretation, we found that genome sizes among RefSeq STIBs tended to be larger on average than genome sizes among MAG-SGBs in all considered environments, even after correcting for MAG incompleteness (Fig. 2). This result confirms and extends a previous finding that uncultured human gut bacteria tend to have significantly smaller genomes when compared to cultured ones^31^. To further examine to what extent size biases in RefSeq can explain the generally higher gene prevalences therein, we adjusted the gene prevalence estimates in RefSeq STIBs for the differences in the size distribution between MAG-SGBs and RefSeq STIBs. The adjustment applied was analogous to those commonly done in stratified demographic surveys with disproportionate sampling of strata, where stratum averages are weighted by each stratum’s proportion in the overall population in order to obtain an unbiased population average^32^ (see Methods for details). We found that this adjustment indeed reduced the discrepancy in gene prevalences between MAG-SGBs and RefSeq STIBs for all environments, with Pearson correlations increasing slightly in all cases and MPRs decreasing substantially towards a value of 1 (Supplemental Fig. S8). Nevertheless, in all environments the MPR remained greater than 1, with the greatest MPR (1.7) found for animal-associated and lake prokaryotes and the smallest MPR (1.3) found for human-associated and ocean prokaryotes. This suggests that size biases partly — but not fully — explain the generally greater gene prevalences estimated for RefSeq STIBs compared to MAG-SGBs.

Second, it is in principle possible that our gene detection approach was less effective in MAGs than RefSeq genomes, for example due to the generally lower quality of MAGs, rather than there being true gene prevalence differences between the two datasets. Further, difficulties still exist in fully reconstructing genomes from metagenomes, notably regarding the inclusion of plasmids and genomic islands^33^. These issues could in principle also cause an under-representation of genes in MAGs, compared to genomes from cultures. To examine this possibility, we repeated our gene prevalence estimates for the subset of MAG-SGBs that could be matched to a RefSeq genome, and for the subset of RefSeq genomes matched by a MAG-SGB. By restricting our gene prevalence estimates to these subsets of MAG-SGBs and RefSeq genomes, we eliminated any major differences in species representation between the two datasets, thus focusing on potential differences in gene inclusion/detection efficacy. For these restricted datasets we found a much closer agreement between gene prevalences in MAG-SGBs and RefSeq genomes, with nearly none of the genes exhibiting a statistically significant difference in prevalence and with MPRs being nearly identical to 1 for all environments (0.97≤MPR≤1.03, Supplemental Fig. S9). We thus conclude that difficulties in including plasmids and genomic islands in MAGs, as well as differences in gene detection efficacy between MAGs and RefSeq genomes, are negligible and, in particular, are not a major source of the gene prevalence differences seen between the full datasets.

Third, RefSeq STIBs may be truly biased towards clades that exhibit the genes examined, to an extent beyond that caused by mere genome size biases. Indeed, KEGG is a highly curated, experimentally informed and functionally focused gene database, and it is possible that genes represented in KEGG tend to be of particular industrial, medical or environmental interests; fewer than half of protein-coding genes predicted in MAGs could be assigned to a KEGG ortholog (overview in Supplemental Table S1). These same interests presumably also guide the majority of culturing and whole sequencing efforts. Similarities between gene characterization biases and culturing/genome sequencing biases will inevitably lead to a tendency for RefSeq STIBs to be rich in genes catalogued in KEGG, even when controlling for genome sizes; we henceforth refer to this mechanism as “intentional” biases. Further, mainstream culturing approaches undoubtedly cause additional unintended trait biases, by favoring fast growers or generalists and disfavoring organisms that depend on syntrophic partners to survive. Genes responsible for (or at least correlating with) traits favored by typical culturing approaches will tend to be more prevalent in cultured species than among prokaryotes in general, and will thus be more likely to be characterized and included in databases such as KEGG. In other words, KEGG could be “unintentionally” biased towards genes that correlate with traits that facilitate culturing. That said, we mention that some genes exhibited lower prevalences among RefSeq STIBs than among MAG-SGBs, suggesting that common culturing approaches may also bias against some genes.

To tease apart intentional from unintentional gene prevalence biases in KEGG, we repeated our analyses with an annotation-independent gene database, the evolutionary gene genealogy of non-supervised orthologous groups (eggNOGs^34^). If biases in KEGG are mostly intentional (as defined above), then one would expect eggNOGs to display a much weaker over-prevalence in RefSeq STIBs than KEGG orthologs do (after adjusting for genome size distributions). In contrast, if biases in KEGG are mostly unintentional (as defined above), then one would expect eggNOGs to display a similar over-prevalence in RefSeq-STIBs as KEGG orthologs do. We found that, when adjusting for genome size distributions, eggNOG MPRs were substantially smaller than KEGG MPRs in 3 out of 6 environments (human, other animals and soil), dropping as low as 0.97 for human-associated species and 1.1 for other animals (Supplemental Fig. S11). This suggests that in these environments the biases in KEGG are to a great extent intentional. In contrast, for bioreactors, ocean and lakes, eggNOG MPRs were greater than KEGG MPRs, suggesting that in these environments the biases in KEGG are largely unintentional and driven by current culturing abilities. That said, further research is needed to better understand the roles of intentional and unintentional biases in the composition of KEGG and other gene databases.

**Fig. 3:**
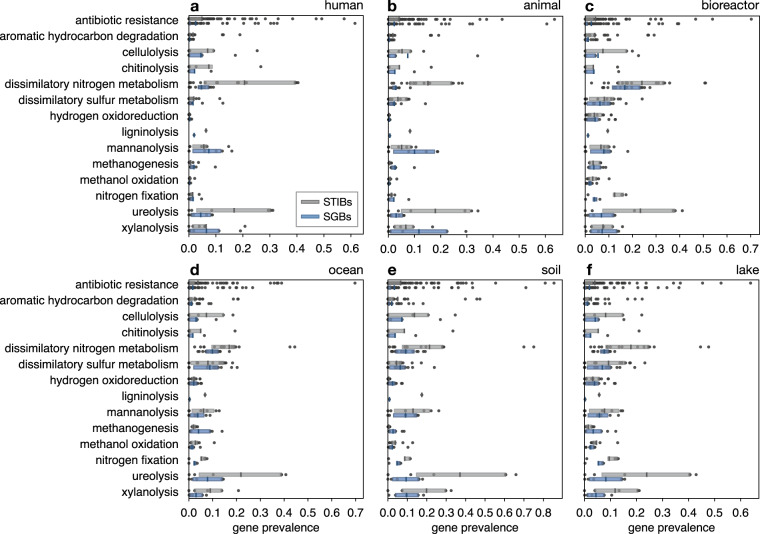
Gene prevalences for selected metabolic functions (MAG-SGBs vs RefSeq STIBs). Box-plots of estimated gene prevalences based on MAG-SGBs and RefSeq STIBs, for selected functions of particular ecological, industrial or medical interest, separately in each environment. Gene prevalences refer to the populations represented by the MAGs and STIBs, i.e., correcting for genome incompleteness. Each box represents a specific function, each point represents a single gene, vertical bar segments denote mean prevalences (i.e., averaged over all genes associated with a specific function), and boxes span the 2nd and 3rd quartile. In most cases gene prevalences are higher among RefSeq STIBs compared to MAG-SGBs, a notable exception being methanogenesis. For similar figures showing gene prevalences in RefSeq STIBs adjusted for the distribution of genome sizes see Supplemental Fig. S12.

To further illustrate the discovered gene prevalence biases from a functional perspective, we examined genes involved in metabolic functions of particular industrial interest, such as lignin, mannan, xylan and cellulose degradation^35–37^, genes involved in functions of particular environmental interest, such as dissimilatory nitrogen and sulfur metabolisms and methanogenesis, as well as genes conferring antibiotic resistance (Fig. 3). For almost all of these functions and regardless of environment, the mean gene prevalences in RefSeq STIBs were considerably higher than in MAG-SGBs. One notable exception was methanogenesis, which was substantially underrepresented in RefSeq STIBs for all environments except bioreactors (where the difference was only minor). This later observation is consistent with the fact that methanogens have been historically difficult to culture^38–40^, and suggests than methanogenesis is a much more common trait in prokaryotes in natural environments than one would expect based on reference genomes.

### Gene-dependent coverage biases

To more precisely quantify the biases for or against various genes in RefSeq, we estimated the conditional probability that a prokaryotic species is covered by (i.e., represented in) RefSeq given that it either has or does not have a given gene. We henceforth denote these two conditional probabilities by *q*_1_ and *q*_0_, respectively. If a gene is neither biased for nor against, we expect *q*_0_ = *q*_1_, while a bias for or against organisms exhibiting the gene would imply *q*_1_ > *q*_0_ or *q*_1_ < *q*_0_, respectively. We estimated *q*_1_ and *q*_0_ separately for each gene by fitting a probabilistic model that accounts for MAG incompleteness, gene presence/absence across MAG-SGBs and matches between MAG-SGBs and RefSeq genomes at ≥95% ANI. To facilitate comparisons between genes and between environments, we considered a composite variable that we termed “coverage bias”, denoted *β* and computed using the estimated *q*_0_, *q*_1_ (see Eq. 4 in Methods for details). The coverage bias is always between −1 and 1, with negative values implying a bias against a gene, positive values implying a bias towards a gene and zero implying no bias (*q*_0_ = *q*_1_). A useful property of *β* is that it only depends on the ratio *q*_1_/*q*_0_ but not on the overall coverage of MAG-SGBs in RefSeq nor on a gene’s prevalence (*α*). We mention that *β* could not be reliably estimated for all genes, since some genes were too rare (*α* ≈ 0) or too prevalent (*α* ≈ 1) for estimating the conditional probabilities *q*_1_ or *q*_0_, respectively.

**Fig. 4:**
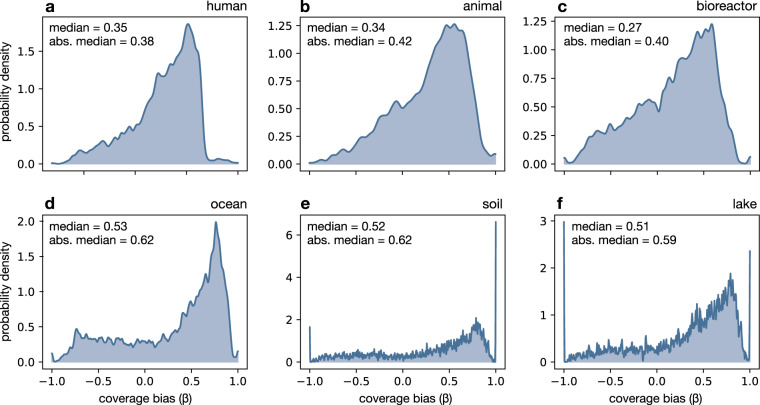
Distribution of gene-specific coverage biases. (**a**) Distribution of gene-specific coverage biases (*β*) for human-associated MAG-SGBs, i.e., biases in the probability of matching a RefSeq genome at ≥95% ANI conditioned on the organism having or lacking a specific gene (KEGG ortholog). For any given gene, a positive bias implies that the probability of an SGB matching a RefSeq genome is greater when the gene is present and smaller when the gene is absent (and vice versa for negative biases). In particular, a bias of +1 implies that the probability of matching a RefSeq genome is zero when the gene is absent. Note that gene presence/absence refers to the population represented by a MAG-SGB, i.e., correcting for MAG incompleteness. The distribution was computed using a kernel density estimate over all considered genes. (**b**–**f**) Similar to (**a**), but for alternative environments. The median bias *β* as well as the median absolute bias (median |*β*|), are written in each figure. Observe that in each environment the majority of coverage biases are positive. A summary of coverage biases for each environment, including kernel density bandwidths, is given in Supplemental Table S4.

We found that in all environments a substantial fraction (50–82%) of considered genes exhibited a statistically significant coverage bias (*β*≠0, *P* < 0.05), with the clear majority of significant coverage biases being positive (Fig. 4 and Supplemental Table S4). Accordingly, the median coverage bias across all genes was positive for all environments (0.27–0.53). This pattern is consistent with our previous observation that gene prevalences tend to be inflated in RefSeq STIBs. In fact, for almost all environments (except human) the distribution of coverage biases exhibited a clear spike at a value of *β* = 1 (i.e., *q*_0_ = 0). For example, among soil-associated prokaryotes 133 out of 4335 considered genes (e.g., *carA*, *gpsA*, *rimP*, *gidB*) exhibited a coverage bias of 1 (details in File KOfam_gene_prevalences_and_biases.tsv.gz on Figshare^41^). Hence, the absence of these genes strongly reduces the probability of a species being represented in RefSeq. That said, we point out that we also found many genes with a significant negative coverage bias (e.g., 14% of genes for soil), which means that species exhibiting these genes are underrepresented in RefSeq. When measured in terms of the median absolute coverage bias (median |*β*|), we observed the strongest coverage biases for the ocean and soil (median |*β*| = 0.62) and the weakest coverage biases for humans (0.38) and bioreactors (0.40). This suggests that existing culturing and genome sequencing pipelines impose weaker trait biases for human- and bioreactor-associated taxa than for ocean- and soil-associated taxa. This observation is perhaps not surprising, given the generally stronger medical and industrial relevance of the first two groups, which probably increases the motivation to culture a broader spectrum of organisms. We also found that coverage biases varied strongly between environments, with the smallest consistency (in terms of the Pearson correlation) seen between humans and soil (*r* = 0.41) and between humans and the ocean (*r* = 0.44, Supplemental Fig. S15).

**Fig. 5:**
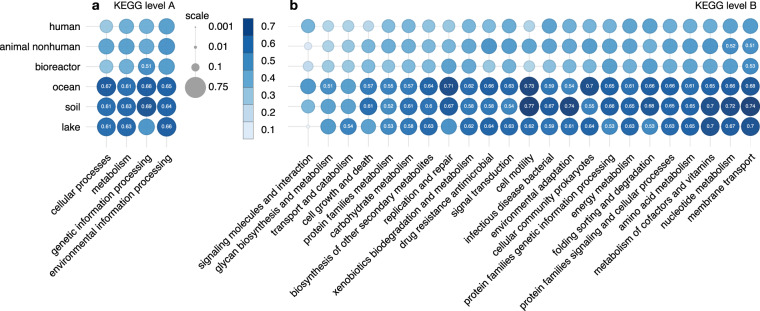
Median absolute coverage biases by environment and gene category. Circle chart of median absolute coverage bias (median |*β*|) for each environment and various gene categories of particular interest (defined according to the KEGG hierarchy, levels A and B). The size and color darkness of each circle are proportional to the median |*β*|. Values above 0.5 are inscribed in the circles. Gene categories are sorted by increasing mean value. Ocean and soil generally exhibit the strongest coverage biases. For a similar graphic showing median coverage biases (median β) see Supplemental Fig. S14.

Strong differences were also observed between different gene categories, as defined by the KEGG KO hierarchy (Fig. 5 and Supplemental Fig. S15). Genes associated with membrane transport, cell motility and nucleotide metabolism generally exhibited the highest median |*β*|, although the precise order depended on the environment considered (also note that only a subset of particularly interesting gene categories was included in this comparison). For ocean and soil, which exhibited the highest median |*β*|, the two considered gene categories with highest median |*β*| were cell motility and membrane transport (median |*β*| between 0.68 and 0.77). This suggests that traits related to cell motility and membrane transport are particularly strong predictors of culturing success in these two environments.

## Discussion

We have analyzed the distribution of thousands of genes across a large culture-independent collection of prokaryotic genomes, and have quantified gene-specific biases in RefSeq reference genomes. Given the general close association between prokaryotic culturing and the existence of a reference genome in RefSeq, as well as the close association between gene content and functional traits in prokaryotes, we expect that our conclusions largely translate to trait biases in prokaryotic cultures. We mention that MAG datasets may exhibit their own biases, for example towards more abundant organisms, or against organisms with multiple hard-to-assemble regions such as 16S SSU rRNA genes, and these biases could in principle influence some of the patterns reported here. However, there is little reason to believe at this point that these biases are driven by traits in a manner that is consistent across locations; in other words, we expect that these biases will tend to average out across locations, and thus not be a substantial driver of the trait biases in RefSeq relative to MAGs.

Genome-resolved metagenomics is greatly accelerating the discovery of novel microbial diversity^19,20,42,43^. Notwithstanding these breakthroughs, it should be remembered that culturing and associated whole genome sequencing remain essential tools for understanding the physiology and ecology of prokaryotes, and for decoding their genotype-phenotype mapping^1,44^. Our results suggest that the current culturing and associated whole genome sequencing efforts are heavily biased towards or against a variety of traits, particularly among ocean- and soil-associated prokaryotes. These biases lead to a distorted distribution of annotated genes/traits in reference databases, which in turn compromises the ecological conclusions one may draw from these distributions. These biases will also inevitably influence gene content and trait predictions for novel clades, for example in 16S rRNA amplicon sequencing studies, when these predictions are performed based on reference genome sets (as is common practice, e.g.^13,45^). In fact, the estimated coverage biases of many genes vary strongly between environments (Supplemental Fig. S15), suggesting that site-specific trait databases and statistical corrections may be needed for accurate trait estimation in novel clades. In addition, coverage biases may also delay the discovery of new industrially useful clades, such as novel methanogens for biofuel production, which we showed were severely underrepresented in RefSeq for all natural environments examined. The gene prevalences and coverage biases estimated in this study (provided as file KOfam_gene_prevalences_and_biases.tsv.gz on Figshare^41^) could help alleviate these issues in the future. For example, the conditional coverages (*q*_0_ and *q*_1_) estimated here can be used to correct for biases in phylogenetic trait prediction algorithms^16^, and help steer future culturing efforts towards under-explored traits.

## Methods

### MAGs

MAGs from 203 distinct studies were either downloaded from NCBI GenBank or from other locations provided by published studies^46–212^. Only studies in which all MAGs were obtained from a similar environment or in which the environment was specified individually for each MAG were considered. For efficiency in MAG collection, we focused on studies with at least 30 MAGs. Studies focusing on a single clade (e.g., *Thaumarchaeota*) or trait (e.g., only methanogens) were omitted. An overview of included studies, including accession numbers and publication references, is provided in file project_metadata.tsv on Figshare^41^. With the exception of one study (“Genomes from Earth’s Microbiomes” or GEM^137^,), all other studies focused on specific environments. The GEM study itself comprised MAGs recovered from a multitude of environments across the world. Ten GEM MAGs were omitted because of missing taxonomic information. MAG qualities were determined based on the presence or absence of multiple universal single-copy genes using checkM2 v0.1.3^213^. MAGs with completeness below 80% or contamination above 5% were omitted; thus 116,884 MAGs were kept for our analyses. An overview of completeness and contamination levels of kept MAGs is shown in Supplemental Fig. S1. We mention that these quality criteria do not exactly match commonly suggested conventions for “high” or “medium” quality MAGs^214^. Instead, the chosen thresholds are based on a reasonable balance between dropping too many MAGs and ensuring sufficient quality in the remaining MAG set. For example, a completeness of at least 90% suggested for “high quality” MAGs by^214^ would be too stringent for some environments such as soil, while a completeness of at least 50% suggested for “medium quality” MAGs would be too low for reliably estimating gene prevalences.

The full size of the genome represented by a MAG (i.e., correcting for MAG incompleteness) was estimated by dividing the size of the MAG (in base pairs) by the completeness determined using checkM2. The taxonomic identities of MAGs were determined using the GTDB-Tk v1.4.1 workflow classify_wf^215^, except for the GEM MAGs for which taxonomic identities were already provided by the original study. All software mentioned above were used with default options unless mentioned otherwise. An overview of represented prokaryotic phyla is shown in Supplemental Fig. S2.

MAGs were associated with various (not necessarily mutually exclusive) environments of interest based on the description in the publication associated with each study (if available), or based on the project description on GenBank, or — in the case of GEM genomes — based on the metadata table provided by the GEM study^137^. MAGs classified as human- and other animal-associated were explicitly excluded from the other environmental categories. Environments associated with each MAG are listed in MAG_metadata_QF.tsv.gz on Figshare^41^.

To avoid species-level redundancies within the MAG dataset, we clustered MAGs into species genome bins (SGBs) at an ANI cutoff of 95% using a similar approach as described by^216^, separately for each environment. Specifically, ANIs between all MAGs from a given environment were calculated using mash v2.3^217^, with sketch size 5000 and otherwise default options. The average nucleotide divergence (AND) between any two MAGs was defined as 1-ANI. Bifurcating trees were constructed based on pairwise ANDs and using the hierarchical clustering algorithm implemented in the R package fastcluster v1.2.3 (function hclust with average linkage)^218^. For computational efficiency, prior to clustering, MAGs were split into smaller disjoint subsets of moderately to closely related MAGs, based on an AND cutoff threshold of 15%. Hierarchical clustering trees were rooted via the midpoint method^219^, using the R package castor v1.7.2^220^. Note that each tip in a tree corresponded to a MAG. Next, tips in the hierarchical clustering trees were grouped into SGBs based on a maximum pairwise distance of 5% AND, using the function collapse_tree_at_resolution in the R package castor^220^. From each SGB, a single representative MAG was kept, chosen to be the MAG with the highest completeness. A total of 29,531 SGBs were thus obtained. An overview of MAGs and SGBs from each environment is given in Supplemental Table S2.

### RefSeq genomes

Genomes were downloaded from the NCBI RefSeq database on October 7, 2021. All genomes whose genome_rep was “Full”, whose gap_fraction was below 0.1, and whose assembly_level was one of “Complete Genome”, “Contig”, “Scaffold”, “Chromosome”, were downloaded. RefSeq genomes were grouped into various (not necessarily mutually exclusive) environments based on the associated biosample’s metadata “geo_loc”, “biosample_organism_name”, “metagenome_source”, “env_local_scale”, “isolation_source” and “isolation_site”, as follows. Genomes whose aforementioned metadata contained any of the words “soil”, “rhizosphere”, “rhizoplane”, “root nodule” or “permafrost” were classified as soil-associated. Genomes whose aforementioned metadata contained any of the words “ocean”, “marine” or “estuary” were classified as ocean-associated. Genomes whose aforementioned metadata contained any of the words or phrases “lake”, “lakewater”, “freshwater sediment”, “freshwater mat” or “pond” were classified as lake-associated. Genomes whose aforementioned metadata contained any of the words or phrases “bioreactor”, “wastewater treatment”, “digester”, “digestor”, “reactor” or “activated sludge” were classified as bioreactor-associated. Genomes either identified as human-associated using FAPROTAX v1.2.4^14^ or whose biosample “host” metadata was “homo sapiens”, were classified as human-associated. Genomes either identified as animal-associated using FAPROTAX v1.2.4^14^ or whose biosample “host” metadata was identified as a metazoan (based on metazoan latin names in the Open Tree of Life v13.4^221^ and a custom list of animal common names), and not already classified as human-associated, were classified as non-human-animal-associated (in this study simply “animal-associated” for brevity). Note that FAPROTAX provides a convenient means to identify human- and animal-associated species, and is used here for the sole reason of increasing the accuracy of the environmental classifications of genomes. Genomes classified as human- and other animal-associated were subsequently excluded from the other environmental categories. A total of 184,131 genomes could be associated with at least one of the above environments. The number of genomes associated with each environment is given in Supplemental Table S2. The environments associated with each RefSeq genome are listed in file RefSeq_genome_metadata_QF.tsv.gz on Figshare^41^.

To avoid species-level redundancies in some of our subsequent analyses, we clustered RefSeq genomes into species-level bins (STIBs) based on their provided species-level taxon identity (species_taxid field). When choosing STIB representatives we prioritized genomes based on their contig-N50 quality metric. An overview of RefSeq genomes and STIBs from each environment is given in Supplemental Table S2.

It is possible that contaminations exist in some RefSeq genomes, with reportedly isolate genomes actually originating from co-cultures, due to the difficulties of growing bacteria (notably Cyanobacteria) axenically^222,223^. Such contaminations, if widespread, could in principle introduce errors in our gene prevalence estimates for RefSeq, although Cyanobacteria only constitute a very small fraction of the RefSeq genomes and we are unaware of any evidence suggesting that such issues are common across RefSeq. Future similar studies could avoid these issues (as well as assembly/binning issues discussed below) by utilizing single-cell amplified genomes^224^.

### Gene detection

Protein-coding genes were predicted for each MAG using prodigal v2.6.3, and were subsequently annotated (matched to KEGG orthologs) using the KOfam Hidden Markov Model database^27^ (release 2020-04-02, comprising 21461 genes) and hmmsearch v3.3.2^225^. A similar approach was used to detect and annotate genes in prokaryotic RefSeq genomes. To reduce computation time, we only annotated a random subset of 137,726 genomes, however all STIB representatives were explicitly included in this subset. Genes not found in any MAG nor any functionally annotated RefSeq genome were omitted from subsequent analyses, as most of these genes are likely eukaryote specific. Thus, a total of 12,454 genes were kept. The number of MAGs, MAG-SGBs, RefSeq genomes and RefSeq STIBs in which each was gene found is listed in file KOfam_gene_prevalences_and_biases.tsv.gz on Figshare^41^. Histograms of the number of MAG-SGBs and RefSeq STIBs in which each gene was detected are shown in Supplemental Figs. S3, S4, respectively. The average number of predicted protein-coding genes per MAG and per genome, as well as the fraction of such genes that could be functionally annotated using KOfam, are listed in Supplemental Table S1.

To examine the role of potential biases in the KEGG database we also matched predicted genes to the eggNOG 5.0 database of orthologous groups^34^, as follows. Protein sequences of all eggNOG orthologs were downloaded from the eggNOG website at http://eggnog5.embl.de/download/eggnog_5.0 (file e5.proteomes.faa.gz). Amino acid sequences predicted in MAGs or RefSeq genomes were then matched to the downloaded protein sequence database using diamond v2.0.15.153^226^, and only hits with an e-value below 10^−10^ were kept. Matched sequences were converted to eggNOG ortholog IDs using a lookup table downloaded from the eggNOG website (file all_members.tsv.gz). For computational tractability, only 50,000 randomly selected eggNOG orthologs were considered for subsequent analysis. Note that the focus of this article is on KEGG orthologs (KOs), hence unless specified otherwise “gene” refers to a KO rather than an eggNOG ortholog.

### Estimating gene prevalences in MAG-SGBs

To estimate the prevalence of a given gene (KEGG ortholog or eggNOG) in populations represented by our MAG-SGB set, i.e., the probability *α* that the population represented by a randomly chosen MAG-SGB exhibits the gene, we proceeded as follows. Throughout the analysis described below, we only considered the single representative MAG of each SGB. We assumed that the probability of a gene being detected in a randomly chosen MAG (using hmmsearch as described earlier) is given by the product *α*·*C*, where *α* is the true prevalence of the gene across species and *C* is the completeness of the MAG. We also assumed that the false positive and false negative detection rate of genes in MAGs are negligible. Hence, if *M*_0_ is the set of MAGs in which the gene was not detected, and *M*_1_ the set of MAGs where the gene was detected, the total likelihood of our dataset (for a given *α*) is given by the product of probabilities:

1 $$L=\prod _{m\in {M}_{0}}\left(1-\alpha {C}_{m}\right)\cdot \prod _{m\in {M}_{1}}\alpha {C}_{m},$$

where *C*_*m*_ is the completeness of the *m*-th MAG. The maximum-likelihood estimate of *α*, denoted $\widehat{\alpha }$, can be found by demanding that the derivative ∂*L*/∂*α* is zero, which is equivalent to the following equation:

2 $$\left|{M}_{1}\right|=\sum _{m\in {M}_{0}}\frac{\widehat{\alpha }{C}_{m}}{1-\widehat{\alpha }{C}_{m}},$$

where is the cardinality of |*M*_1_|. Note that if all MAGs were complete (*C*_*m*_ = 1 for all *m*), the solution to Eq. (2) would simply be $\widehat{\alpha }=\left|{M}_{1}\right|/\left(\left|{M}_{0}\right|+\left|{M}_{1}\right|\right)$, however in reality most MAGs were incomplete, making an analytical solution difficult. Equation (2) was thus solved numerically for $\widehat{\alpha }$ in python, using the bisection method. Confidence intervals were obtained using parametric bootstrapping, i.e., based on gene presences/absences generated randomly across the MAGs according to the above statistical model and the fitted *α*. We mention that, like most bioinformatics analyses in this paper, checkM2 -based completeness estimates may not be fully accurate. Errors in MAG completeness estimates would introduce errors in the gene prevalence estimates, although these errors are suspected to be small based on typical checkM2 errors (mean average error ~3%)^213^. Further, we mention that the MAGs and genomes analyzed were originally generated using a variety of alternative sequencing platforms, assembly and binning tools. This methodological variation could in principle impact contig assembly lengths, which ORFs are included/split on those contigs, the frequency of chimeric contigs, and which contigs get included in each bin, which would by extension impact the recovery of ORFs and the estimation of gene prevalences and coverage biases. That said, our analysis of gene prevalences restricted to MAG-SGBs matched by RefSeq genomes (see main article and Supplemental Fig. S9) indicated that ORF recovery and gene detection efficiency did not noticeably differ between MAG-SGBs and their matched RefSeq genomes.

### Estimating gene prevalences in RefSeq STIBs

Prevalences of genes (KEGG orthologs or eggNOGs) across RefSeq STIBs were estimated using a similar approach as for MAG-SGBs, the main difference being that the completeness of a RefSeq genome was computed based on the associated gap fraction listed in RefSeq (that said, we mention that the gap fraction was negligible for the majority of RefSeq genomes considered). To further examine how the gene prevalence estimates across RefSeq STIBs would change in the absence of any genome size biases (relative to the MAG dataset), i.e., correcting for the distribution of genome sizes, we proceeded as follows. We binned MAG-SGBs based on their estimated full genome size (i.e., correcting for MAG incompleteness) into 0.5 Mbp size intervals or “strata” (i.e., 0–0.5 Mbp, 0.5–1 Mbp, 1–1.5 Mbp, …). Similarly, we binned RefSeq STIBs based on their full genome size (attribute total_length) into the same size intervals, and independently estimated gene prevalences separately for each size interval, i.e, treating each set of STIBs in a size interval as a separate dataset. For each gene, we then computed the weighted average prevalence across all size intervals, weighting each size interval by the number of MAG-SGBs in that interval. This weighting adjustment is commonly performed in demographic surveys in which different strata are sampled at different proportions^32^. For comparisons of gene prevalence estimates between MAG-SGBs and RefSeq STIBs see Fig. 1, and Supplemental Figs. S8–S11.

### Estimating coverage biases

For the following analysis, MAGs have been deduplicated at the SGB level, i.e., we considered only one representative MAG per SGB. We say that a MAG “matches” a RefSeq genome if its ANI to that genome (as calculated using mash) was at least 95%. By “coverage” we mean the probability that a randomly chosen MAG matches a RefSeq genome. To investigate the coverages of MAGs, separately for each environment and depending on the presence or absence of specific genes (KEGG orthologs or eggNOGs), we proceeded as follows. For any given environment, let *q* denote the overall coverage, i.e., the probability that a randomly chosen MAG matches a RefSeq genome. For any given environment and gene, let *q*_0_ and *q*_1_ be the conditional probabilities that a randomly chosen MAG matches a RefSeq genome given that the population represented by the MAG lacked or had the gene, respectively. Note that a gene may be missing from an incomplete MAG even if the represented population had the gene. The two conditional probabilities *q*_0_ and *q*_1_ are a priori unknown, and correspond to the coverage of MAGs in the absence or presence, respectively, of the gene in the represented populations. For example, if *q*_0_ > *q*_1_ then this means that RefSeq is biased towards organisms lacking the gene, whereas if *q*_0_ < *q*_1_ RefSeq would be biased towards organisms having the gene. Note that by mathematical necessity either *q*_0_ ≤ *q* ≤ *q*_1_ or *q*_0_ ≥ *q* ≥ *q*_1_, i.e., *q*_0_ and *q*_1_ cannot be both above or both below the overall coverage *q*. Our first objective was to estimate the *q*_0_ and *q*_1_ based on our MAG dataset. For any given environment and gene, let ${M}_{0}^{0}$ be the set of MAGs in which the gene was not detected and which did not match any RefSeq genome, let ${M}_{0}^{1}$ be the set of MAGs in which the gene was not detected and which did match a RefSeq genome, let ${M}_{1}^{0}$ be the set of MAGs in which the gene was detected but which did not match any RefSeq genome, and let ${M}_{1}^{1}$ be the set of MAGs in which the gene was detected and which did match a RefSeq genome. As before, *C*_*m*_ denotes the completeness of the *m*-th MAG, and *α* denotes the probability that the population represented by a randomly selected MAG had the gene. Hence, for example, the probability of detecting a gene in a randomly chosen MAG together with that MAG matching a RefSeq genome is given by the product *αC*_*m*_*q*_1_. The probability of not detecting the gene in a randomly chosen MAG and that MAG not matching any RefSeq genome is given by the sum of probabilities of two complementary events: either the represented population did not have the gene and its MAG did not match any RefSeq genome (probability (1−*α*)(1−*q*_0_)), or the population did have the gene but the gene was missing from the MAG (due to incompleteness) and the MAG did not match any RefSeq genome (probability *α*(1−*C*_*m*_)(1−*q*_1_)). Similar arguments can be made for all other possible scenarios as well, eventually leading to the following expression for the likelihood of our data:

3 $$\begin{array}{l}L=\prod _{m\in {M}_{0}^{0}}\left[\left(1-\alpha \right)\left(1-{q}_{0}\right)+\alpha \left(1-{C}_{m}\right)\left(1-{q}_{1}\right)\right]\times \prod _{m\in {M}_{0}^{1}}\left[\left(1-\alpha \right){q}_{0}+\alpha \left(1-{C}_{m}\right){q}_{1}\right]\\ \times \prod _{m\in {M}_{1}^{0}}\alpha {C}_{m}\left(1-{q}_{1}\right)\times \prod _{m\in {M}_{1}^{1}}\alpha {C}_{m}{q}_{1}.\end{array}$$

The maximum likelihood estimates ${\widehat{q}}_{0}$ and ${\widehat{q}}_{1}$ were obtained by numerically maximizing the log-likelihood ln(*L*) in python and using the previously obtained maximum-likelihood estimate $\widehat{\alpha }$. To avoid inaccurate estimates of the conditional coverages *q*_0_, *q*_1_, we only considered genes detected in at least 100 MAGs and missing from at least 100 MAGs. Further, optimization of the likelihood failed for a small fraction of genes. Thus, the specific set of genes considered differed somewhat between environments (overview in Supplemental Table S4, details in file KOfam_gene_prevalences_and_biases.tsv.gz on Figshare^41^). For an overview of estimated conditional coverages see Supplemental Fig. S13.

To quantify the strength of bias for or against a gene in a way that facilitates comparison between environments, we defined the “coverage bias” as follows:

4 $$\begin{array}{l}\beta ={\rm{sign}}\left({q}_{1}-{q}_{0}\right)\cdot \left[1-\frac{min\left({q}_{0},{q}_{1}\right)}{max\left({q}_{0},{q}_{1}\right)}\right].\end{array}$$

The coverage bias *β* can also equivalently be written as follows:

5 $$\begin{array}{l}\beta =\left(\begin{array}{cc}1-\frac{{q}_{0}}{q1} & :{q}_{1}\ge {q}_{0}\\ \frac{{q}_{1}}{{q}_{0}}-1 & :{q}_{1}\le {q}_{0}\end{array}\right..\end{array}$$

Observe that *β* is always between −1 and 1, and that a positive (or negative) value implies a bias for (or against) the specific gene. A value of *β* = 0 implies that *q*_0_ = *q*_1_ = *q* and hence an absence of any bias related to the gene. A value of *β* = 1 implies that *q*_0_ = 0, which means that the absence of the gene in an organism makes it improbable that the organism is represented in RefSeq (at ANI ≥95%). On the other extreme, a value of *β* = −1 implies that *q*_1_ = 0, which means that the presence of the gene in an organism makes it improbable that the organism is represented in RefSeq. A useful property of *β* is that it only depends on the ratio *q*_1_/*q*_0_ but not on the overall coverage *q* nor on the gene’s prevalence *α*, thus making it suitable for exploring the effects of the environment on gene-specific coverage biases. For example, if MAGs from populations having the gene are 3 times more probable to match a RefSeq genome compared to MAGs from populations lacking the gene (i.e., *q*_1_ = 3*q*_0_), then $\beta =1-\frac{1}{3}=2/3$ regardless of whether that environment in and of itself is strongly biased for or against in RefSeq, and regardless of the gene’s prevalence in that environment. The two-sided statistical significance of *β* was determined using parametric bootstrapping under the null model of zero bias (*q*_0_ = *q*_1_), i.e., by randomly re-generating gene presences/absences in the MAGs according to the fitted *α* and accounting for MAG completeness while ignoring MAG coverages. We mention that the above estimates are not adjusted for the differences in the genome size distributions in RefSeq-STIBs versus MAG-SGBs. For example, a *q*_1_ greater than *q*_0_ may be partly due to the fact that the presence of a given gene in a species will tend to correlate positively with the species’ genome size (all else being equal), which in turn will correlate positively with the inclusion of the species in RefSeq, thus increasing *q*_1_ relative to *q*_0_. For a summary of coverage biases see Fig. 4, Supplemental Fig. S16 and Supplemental Table S4.

### Kernel density estimates of genome size distributions

Gaussian kernel density estimates of the distribution of MAG full genome sizes (i.e., accounting for MAG incompleteness) or RefSeq genome sizes (attribute total_length) were computed using the KernelDensity function in the python package scikit-learn v1.0.2^227^. The optimal KDE bandwidth was determined separately for each environment, and separately for MAGs and RefSeq genomes, via 5-fold cross-validation using the function GridSearchCV in scikit-learn. The pool of bandwidths considered ranged from 0.001 up to 10 times the total data range. Optimized KDE bandwidths are listed in Supplemental Table S3.

## Supplementary information

Supplementary Information

## Data Availability

All data have been previously published and are publicly available, as described in the Methods. Supplemental files relating to this analysis are available at Figshare^41^: MAG sources are given in file project_metadata.tsv, accession numbers for MAGs (where available) are given in file MAG_metadata_QF.tsv.gz, accession numbers for RefSeq genomes are given in file RefSeq_genome_metadata_QF.tsv.gz, analysis results for each gene (KEGG ortholog) are given in file KOfam_gene_prevalences_and_biases.tsv.gz, a table of all KOs found per MAG is given as file KOfams_vs_MAGs.tsv.gz and a table of all KOs found per RefSeq genome is given as file KOfams_vs_RefSeq_genomes.tsv.gz.

## Code availability

All software used in this paper have been described in the Methods and are freely available online. A copy of our workflow (bash, python, R code files) is also available at Figshare^41^.
