# Supplementary material for: Trait biases in microbial reference genomes
Source: Sci Data. 2023 Feb 9;10:84. doi: 10.1038/s41597-023-01994-7 (PMC9911409; doi:10.1038/s41597-023-01994-7)
Supplement: Supplementary file 1 — Supplementary Information [file 41597_2023_1994_MOESM1_ESM.pdf]

Trait biases in microbial reference genomes
# - Supplementary Information -

Sage Albright[1] & Stilianos Louca[1,2,*]

[1]*Department of Biology, University of Oregon, Eugene, USA*
[2]*Institute of Ecology and Evolution, University of Oregon, Eugene, USA*
[*]Corresponding author

Table S1: **Protein prediction & annotation summaries.** Mean number of protein-coding genes predicted for MAGs and RefSeq genomes using `prodigal`, as well as the average fraction of such genes that could be functionally annotated (matched to a KEGG ortholog), separately for each environment.

environment	mean number of predicted proteins in MAGs	mean fraction annotated in MAGs	mean number of predicted proteins in RefSeq genomes	mean fraction annotated in RefSeq genomes
human	2427	0.445	3871	0.521
animal	2156	0.418	3277	0.462
bioreactor	3035	0.392	4104	0.441
ocean	2469	0.440	3895	0.434
soil	3759	0.361	5645	0.375
lake	2672	0.416	4019	0.441

Table S2: **Overview of MAGs and genomes per environment.** Number of MAGs, MAG-SGBs, RefSeq genomes and RefSeq STIBs analyzed for each environment. Also listed are the number of studies from which MAGs were obtained, the number of RefSeq STIBs included in the KO search, as well as the coverage of MAGs and of MAG-SGBs (fraction of MAGs or MAG-SGBs matched to RefSeq at $\geq$95% ANI, respectively). Note that some of these studies re-analyzed metagenomic sequence data from multiple other sources, and that some studies comprised MAGs from multiple environments.

	Nstudies	MAGs	MAG-SGBs	RefSeq genomes	RefSeq STIBs	MAG coverage	MAG-SGB coverage
human	20	57117	3730	158318	5232	0.693	0.331
animal	30	19694	5745	13744	4597	0.0971	0.0707
bioreactor	35	7790	4463	406	336	0.0621	0.0511
ocean	72	14599	7749	2291	1734	0.0831	0.0547
soil	23	4373	2672	7934	5066	0.122	0.0494
lake	28	13351	5172	1438	1255	0.0278	0.0215

Table S3: **Genome sizes.** Mean genome sizes estimated for MAG-SGBs (correcting for MAG incompleteness) and RefSeq STIBs, separately for each environment (in Mbp). Also listed are the optimal kernel density estimate (KDE) bandwidths, optimized via cross-validation and used to compute the density estimates in Fig. 1.

environment	MAG-SGB mean genome size	RefSeq STIB mean genome size	MAG-SGB KDE bandwidth	RefSeq STIB KDE bandwidth
human	2.5	3.8	0.25	0.19
animal	2.6	3.5	0.13	0.51
bioreactor	3.7	4.4	0.14	0.73
ocean	2.9	4.2	0.13	0.46
soil	3.9	6.1	0.28	0.45
lake	3.1	4.4	0.09	0.44

Table S4: **Summary of coverage biases (MAG-SGBs).** Fraction of genes (KEGG orthologs) exhibiting a positive coverage bias ($\beta > 0$), a negative coverage bias ($\beta < 0$), a statistically significant positive coverage bias, or a statistically significant negative coverage bias. Also listed are the numbers of genes considered (Ngenes, see Methods for inclusion criteria), the median bias (median β), the median absolute bias (median $|\beta|$) and kernel density estimate (KDE) bandwidths used for Fig. 4 (optimized via cross-validation).

environment	Ngenes	$\beta > 0$	$\beta < 0$	$\beta > 0$ significant	$\beta < 0$ significant	median β	median $\|\beta\|$	KDE bandwidth
human	3701	0.82	0.18	0.72	0.10	0.35	0.38	0.019
animal	4062	0.77	0.23	0.55	0.079	0.38	0.42	0.030
bioreactor	5008	0.69	0.31	0.38	0.12	0.27	0.40	0.021
ocean	5551	0.74	0.26	0.62	0.17	0.53	0.62	0.011
soil	4335	0.73	0.27	0.54	0.14	0.52	0.62	0.0020
lake	4952	0.77	0.23	0.44	0.08	0.51	0.59	0.0026

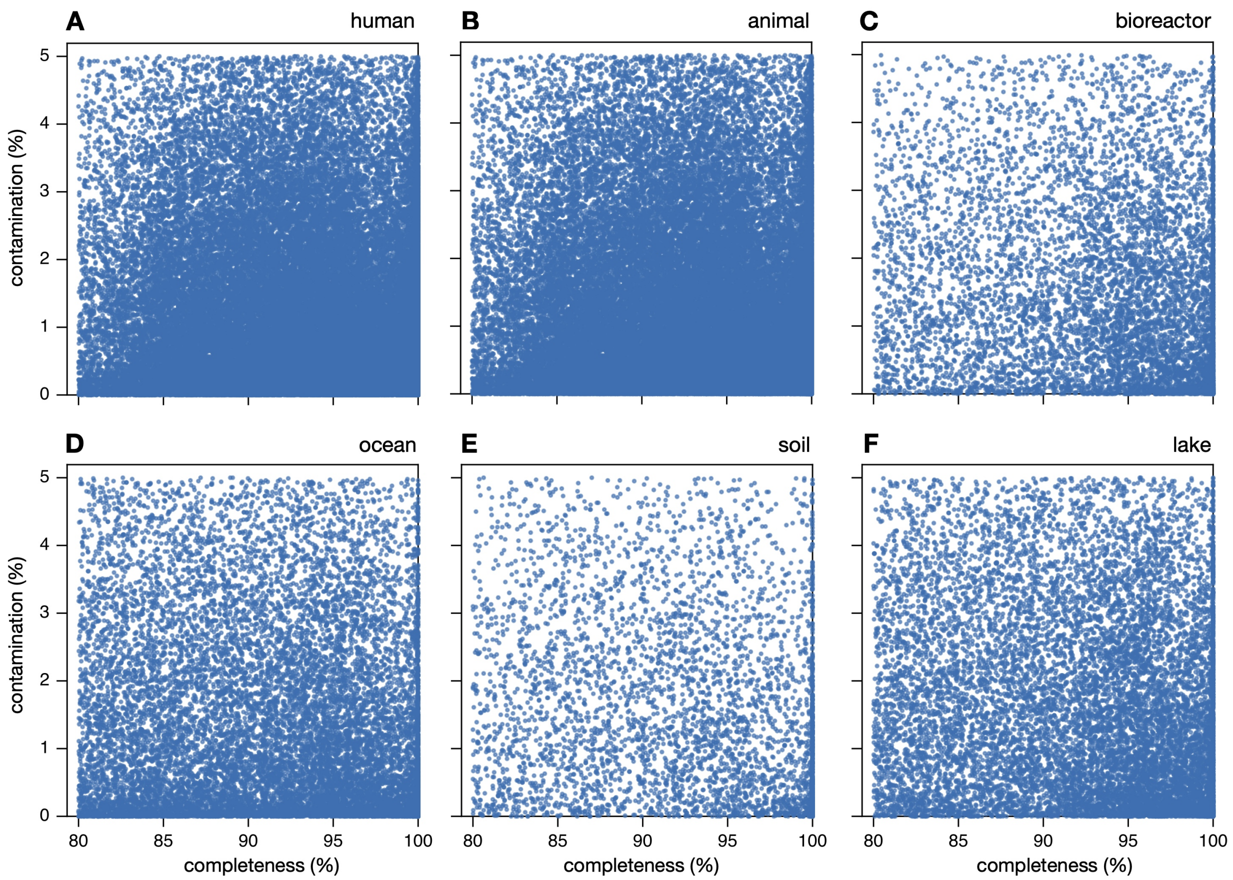

Figure S1: **MAG completeness and contamination.** Estimated completeness (horizontal axis) and contamination (vertical axis) for every MAG considered and meeting our quality criteria (one point per MAG), separately for each environment. For numbers of MAGs and SGBs per environment see Supplemental Table S2.

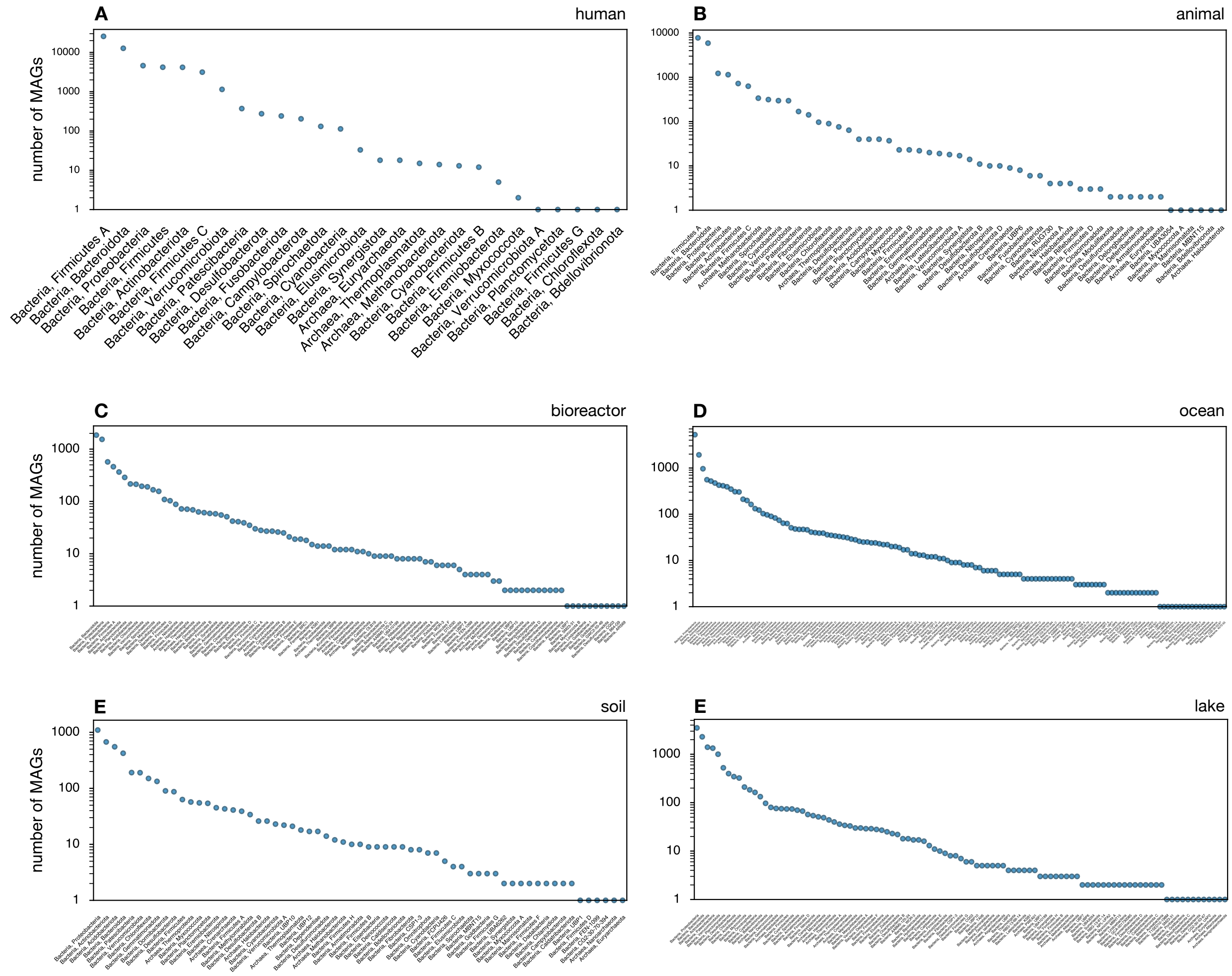

Figure S2: **Taxonomic coverage of MAGs.** Number of MAGs analyzed per phylum, separately for each environment.

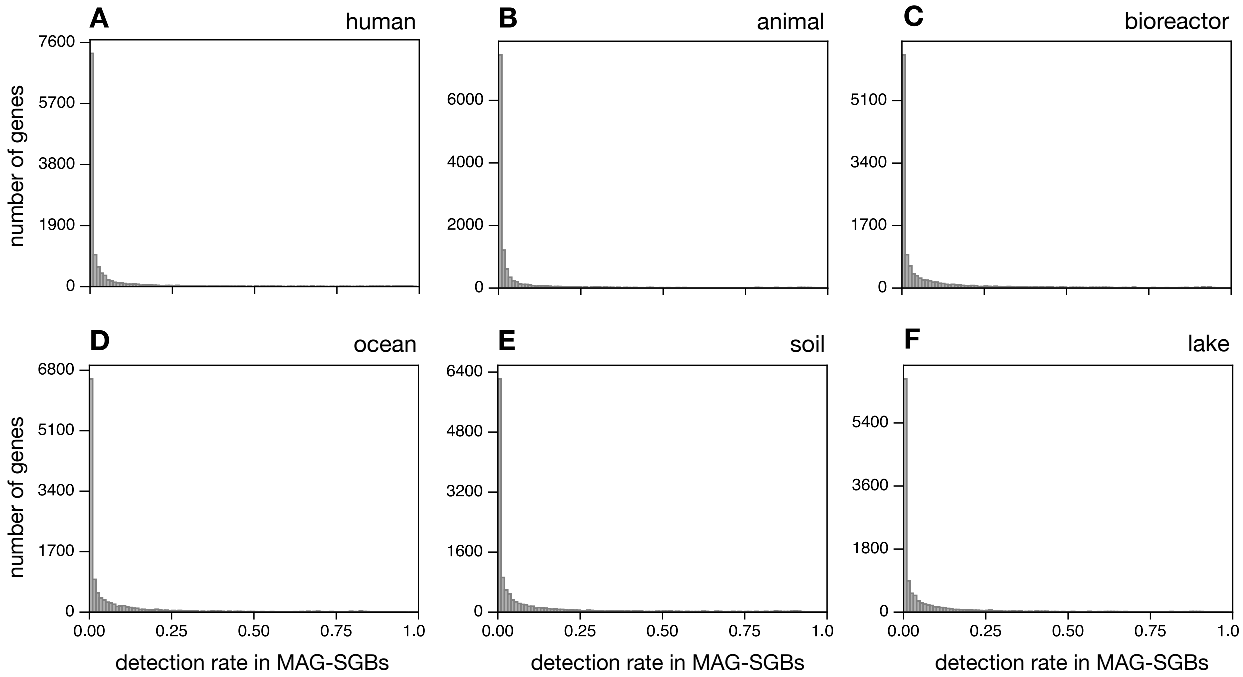

Figure S3: **Distribution of gene detection rates in MAG-SGBs.** Histogram of the number of MAG-SGBs in which each gene (KEGG ortholog) was found, separately for each environment. For estimated true gene prevalences (i.e., correcting for MAG incompleteness) see Supplemental Figure S5.

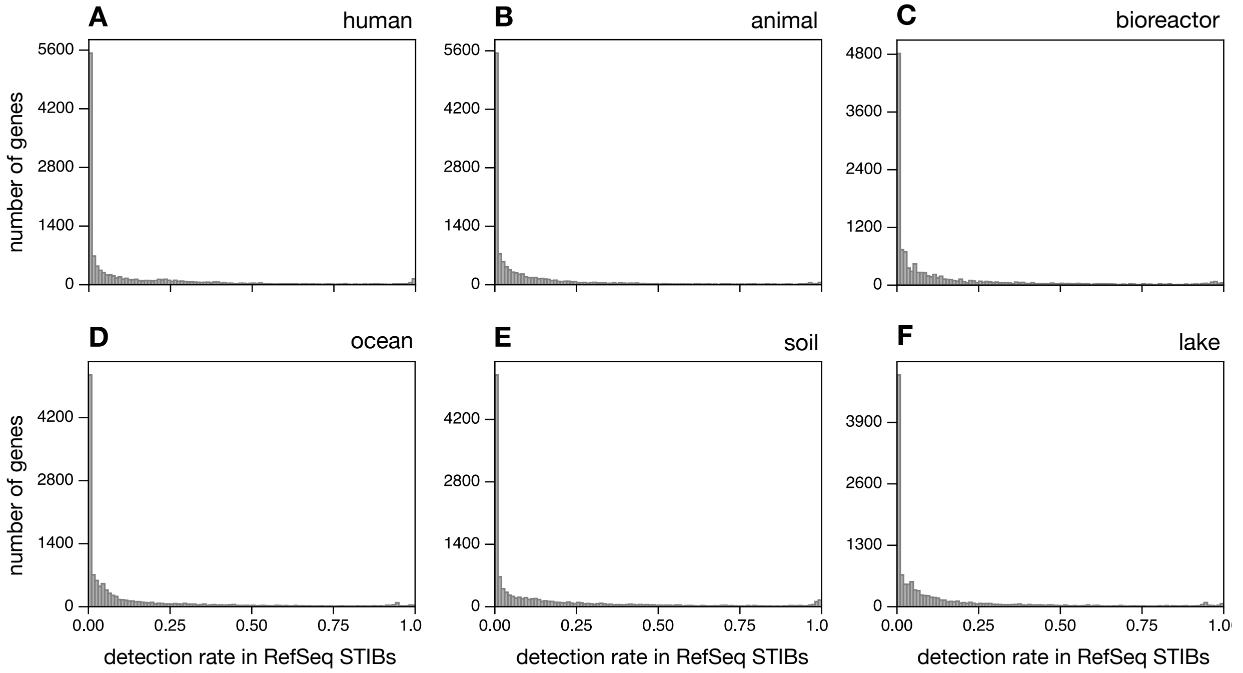

Figure S4: **Distribution of gene detection rates in RefSeq STIBs.** Histogram of the number of RefSeq STIBs in which each gene (KEGG ortholog) was found, separately for each environment.

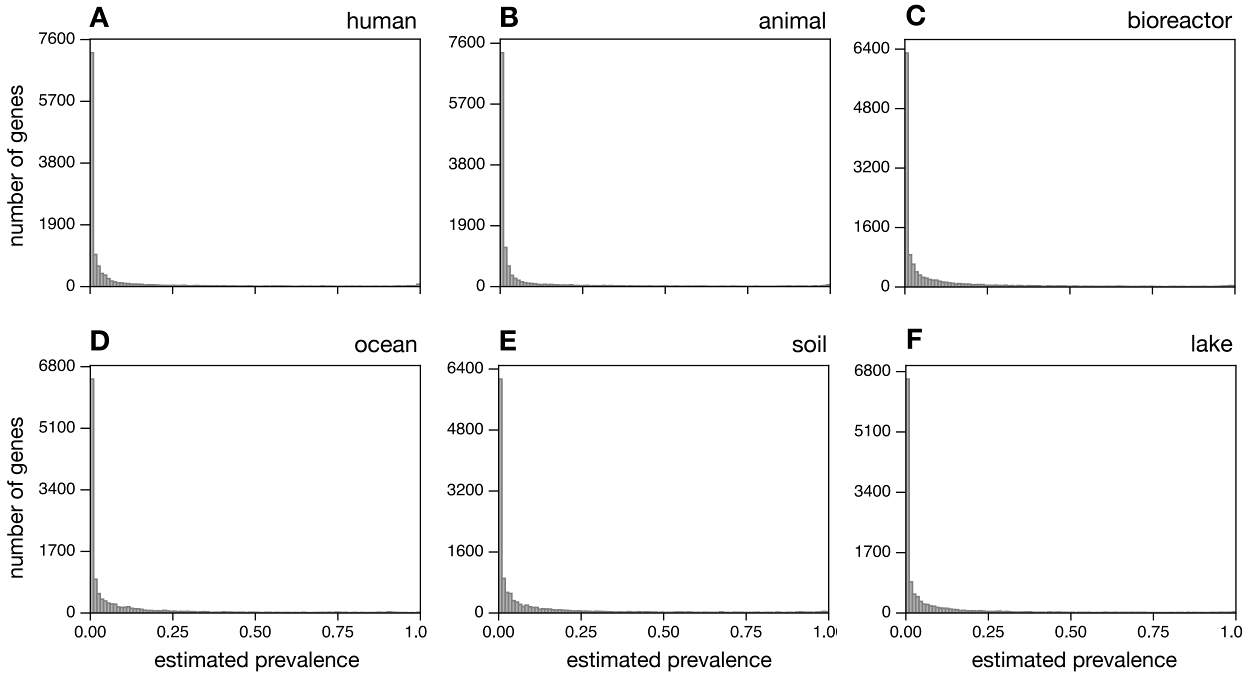

Figure S5: **Distribution of estimated gene prevalences based on MAG-SGBs.** Histogram of the estimated gene (KEGG ortholog) prevalences for MAG-SGBs, correcting for MAG incompleteness and separately for each environment. For gene detection rates without correcting for MAG incompleteness, see Supplemental Figure S3.

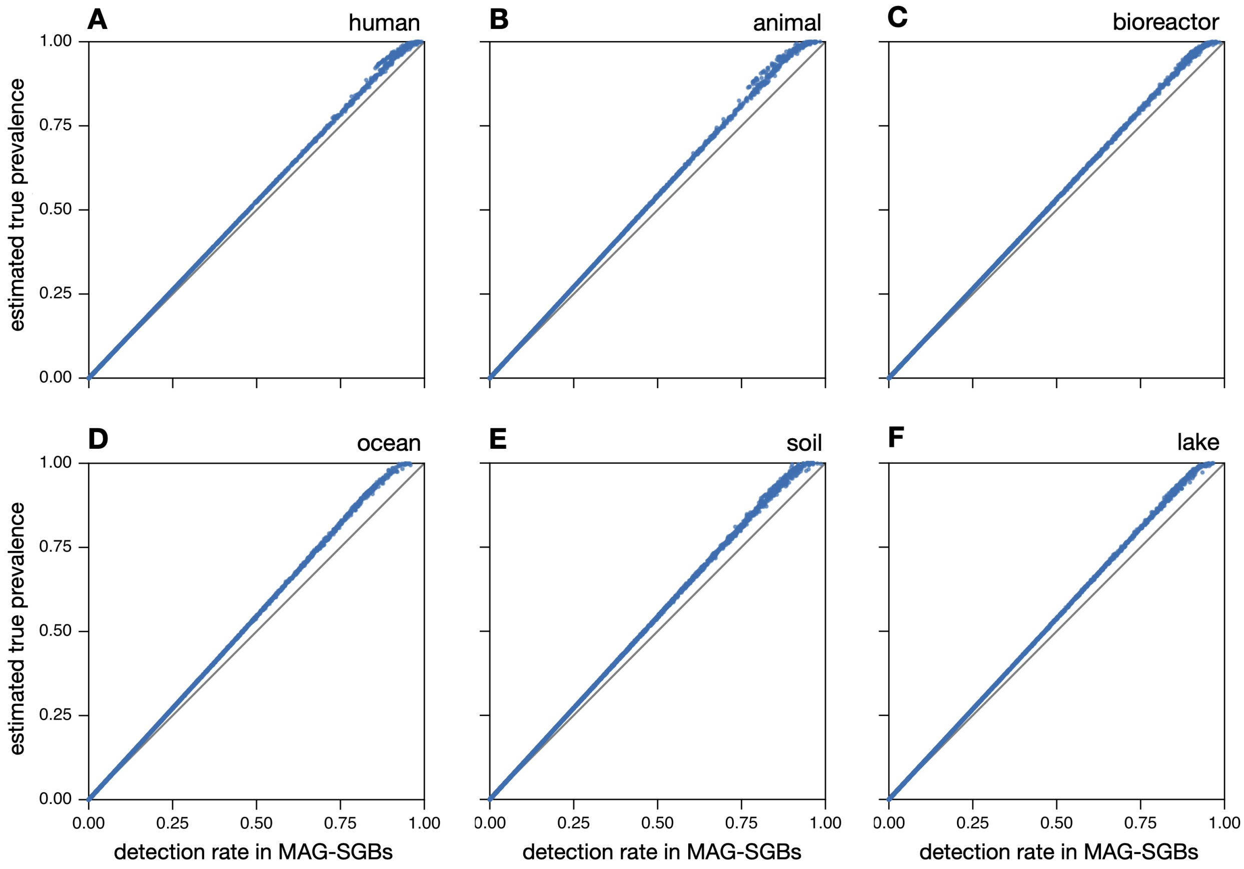

Figure S6: **Gene detection rates vs estimated prevalences (MAG-SGBs).** Detection rates of genes (KEGG orthologs) in MAG-SGBs (fraction of SGB-representative MAGs in which a gene was found, horizontal axes) compared to the estimated true prevalences of genes in SGBs (fraction of represented organisms estimated to exhibit a gene, correcting for MAG incompleteness, vertical axes), separately for each environment (one point per gene, one figure per environment). The diagonal is shown for reference. Observe that the detection rates are generally lower than the estimated true gene prevalences, since most of the analyzed MAGs were incomplete.

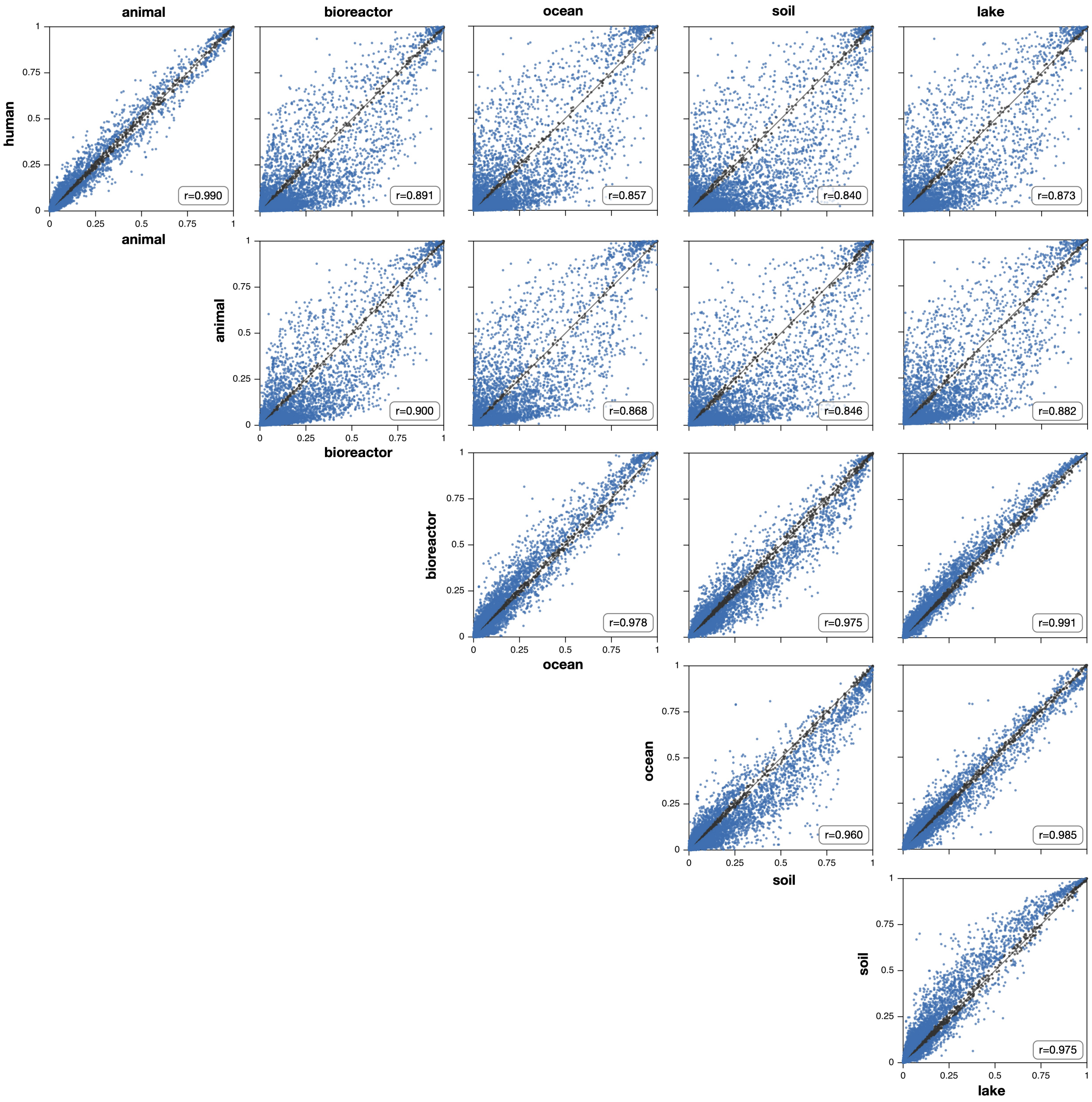

Figure S7: **Estimated gene prevalences based on MAG-SGBs, compared between environments.** Upper left plot: Estimated gene (KEGG ortholog) prevalences for MAG-SGBs (correcting for MAG incompleteness) among prokaryotes associated with humans (vertical axis) and among prokaryotes associated with other animals (horizontal axis, one point per gene). Blue points correspond to genes whose estimated prevalences are significantly different between the compared environments (i.e., the 95% confidence intervals of the two estimates do not overlap), while black points correspond to genes whose prevalences are not significantly different. All other plots: Comparisons between other pairs of environments. Diagonal lines are shown for reference. Pearson correlation coefficients (r) are written in each plot; all correlations were highly significant based on a permutation test ($P<0.001$).

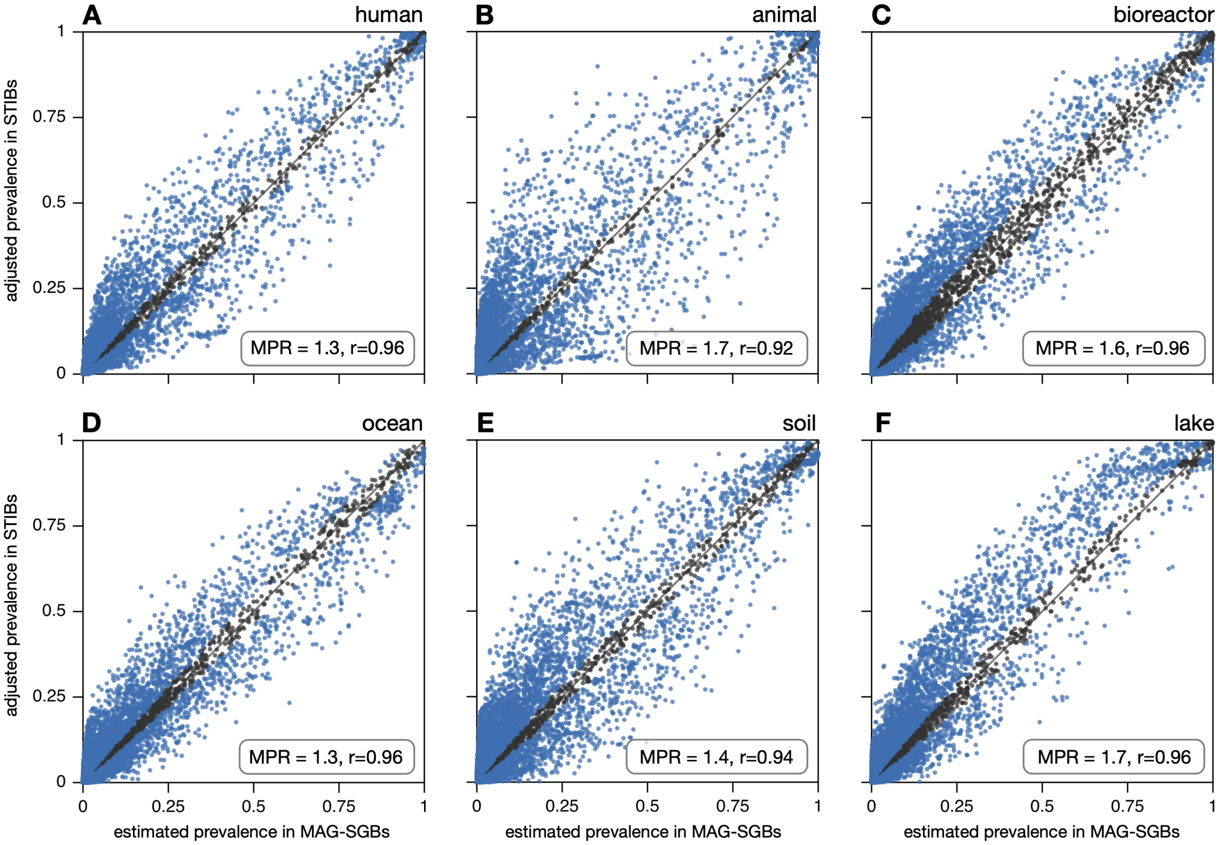

Figure S8: **Gene prevalences (MAG-SGBs vs RefSeq-STIBs, size-adjusted).** Estimated gene (KEGG ortholog) prevalences in MAG-SGBs (horizontal axes) compared to prevalences in RefSeq STIBs adjusted for the distribution of genome sizes (vertical axes), separately for SGBs/STIBs associated with (A) humans, (B) other animals, (C) bioreactors, (D) ocean, (E) soil and (E) lakes. Every dot represents a distinct gene (KEGG Ortholog). Blue dots denote genes whose prevalence is statistically significantly different in SGBs compared to STIBs, while black dots denote genes whose prevalence is not statistically significantly different. The diagonal is shown for reference. The median prevalence ratio (MPR, prevalence in STIBs divided by the prevalence in SGBs, median taken across genes) and the Pearson correlation coefficient (r) are written in each plot; all correlations were highly significant based on a permutation test ($P<0.001$). For similar figures restricted to MAG-SGBs that could be matched to a RefSeq genome, see Supplemental Fig. S9.

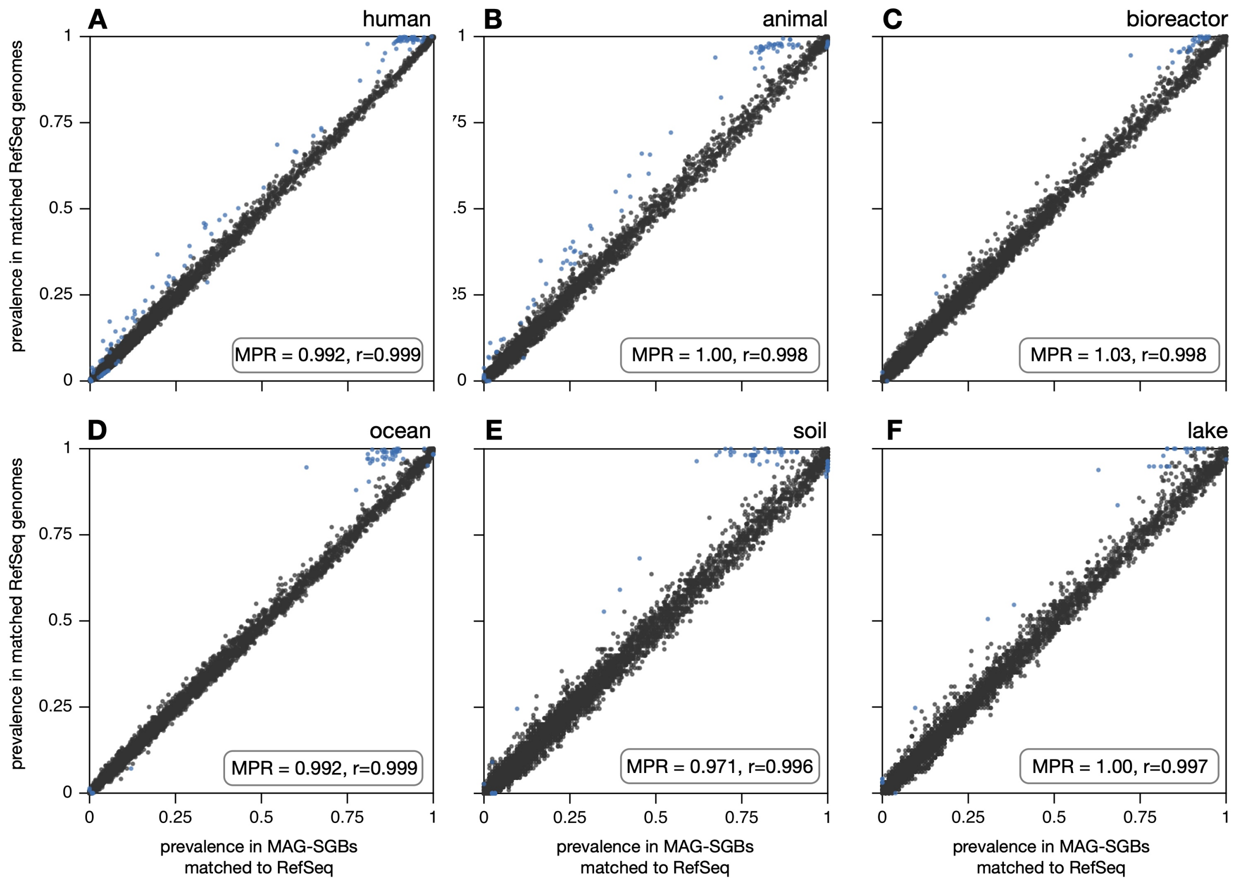

Figure S9: **Gene prevalences (MAGs-SGBs vs RefSeq, matches only).** Gene (KEGG ortholog) prevalences in MAG-SGBs that could be matched to a RefSeq genome at $\geq$95% ANI (horizontal axes) compared to gene prevalences in the matching RefSeq genomes (vertical axes), correcting for MAG and genome incompleteness, separately for SGBs associated with (A) humans, (B) other animals, (C) bioreactors, (D) ocean, (E) soil and (E) lakes. Every dot represents a distinct gene (KEGG Ortholog). Blue dots denote genes whose estimated prevalence is significantly different in the SGBs compared to the matching RefSeq genomes (i.e., the 95% confidence intervals of the two estimates do not overlap), while black dots denote genes whose prevalence is not significantly different. The diagonal is shown for reference. The median prevalence ratio (MPR, prevalence in the RefSeq genomes divided by the prevalence in the SGBs, median taken across genes) and the Pearson correlation coefficient (r) are shown in each plot; all correlations were highly significant based on a permutation test (P<0.001). Abbreviations: MAG, metagenome-assembled genome; SGB, species genome bin; ANI, average nucleotide identity.

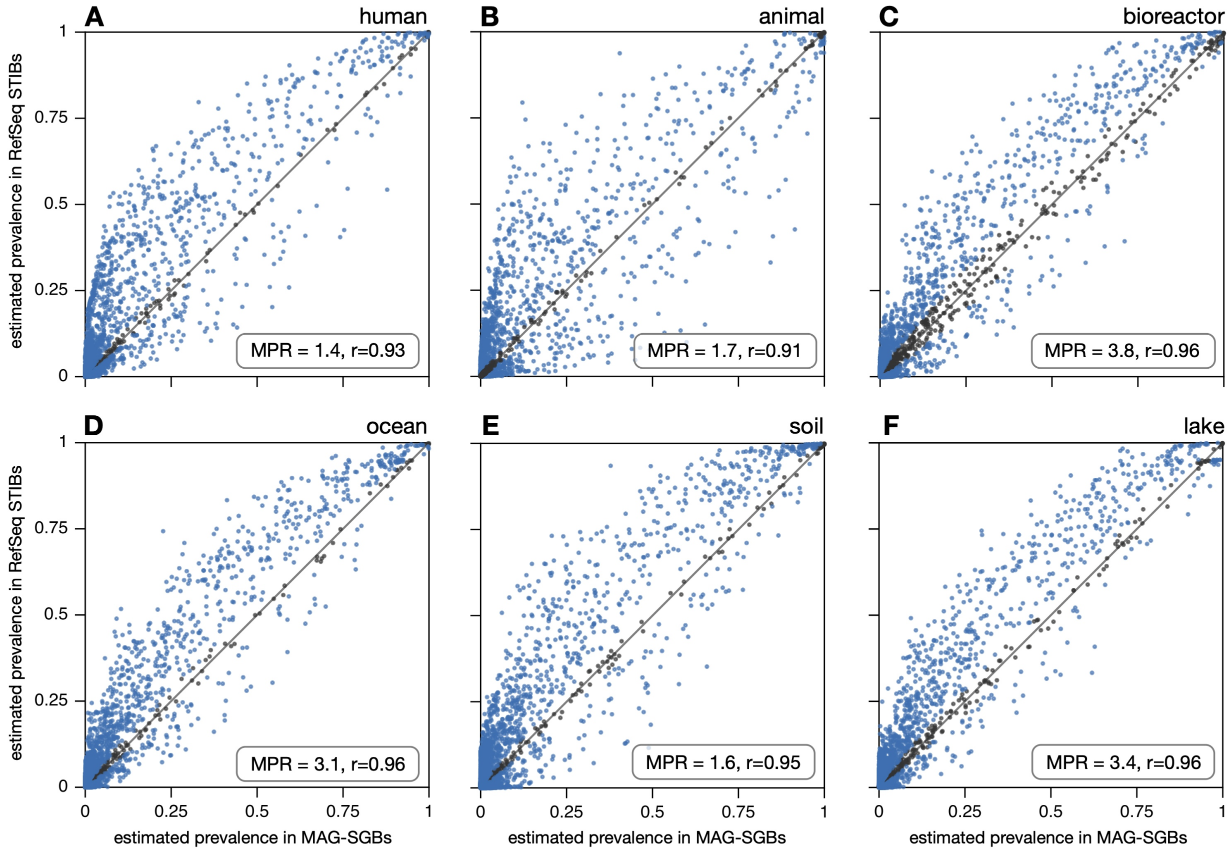

Figure S10: **eggNOG prevalences (MAG-SGBs vs RefSeq-STIBs).** Estimated eggNOG ortholog prevalences in MAG-SGBs (horizontal axes) compared to prevalences in RefSeq STIBs (vertical axes), separately for SGBs/STIBs associated with (A) humans, (B) other animals, (C) bioreactors, (D) ocean, (E) soil and (E) lakes. Every dot represents a distinct eggNOG gene ortholog. Prevalence estimates account for genome incompleteness. Blue dots denote genes whose prevalence is statistically significantly different in SGBs compared to STIBs, while black dots denote genes whose prevalence is not statistically significantly different. The diagonal is shown for reference. The median prevalence ratio (MPR, prevalence in STIBs divided by the prevalence in SGBs, median taken across genes) and the Pearson correlation coefficient (r) are written in each plot; all correlations were highly significant based on a permutation test (P<0.001).

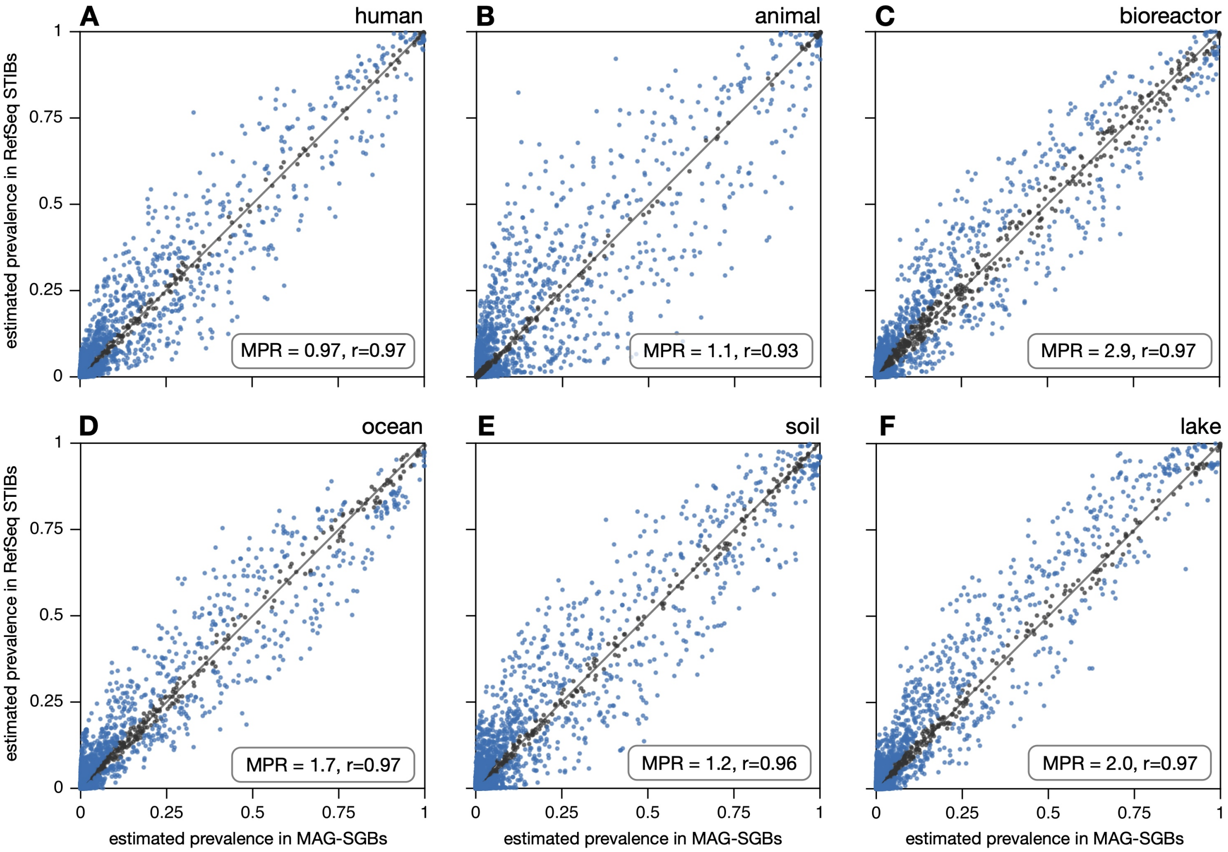

Figure S11: **eggNOG prevalences (MAG-SGBs vs RefSeq-STIBs, size-adjusted).** Estimated eggNOG ortholog prevalences in MAG-SGBs (horizontal axes) compared to prevalences in RefSeq STIBs adjusted for the distribution of genome sizes (vertical axes), separately for SGBs/STIBs associated with (A) humans, (B) other animals, (C) bioreactors, (D) ocean, (E) soil and (E) lakes. Every dot represents a distinct eggNOG gene ortholog. Prevalence estimates account for genome incompleteness. Blue dots denote genes whose prevalence is statistically significantly different in SGBs compared to STIBs, while black dots denote genes whose prevalence is not statistically significantly different. The diagonal is shown for reference. The median prevalence ratio (MPR, prevalence in STIBs divided by the prevalence in SGBs, median taken across genes) and the Pearson correlation coefficient (r) are written in each plot; all correlations were highly significant based on a permutation test (P<0.001).

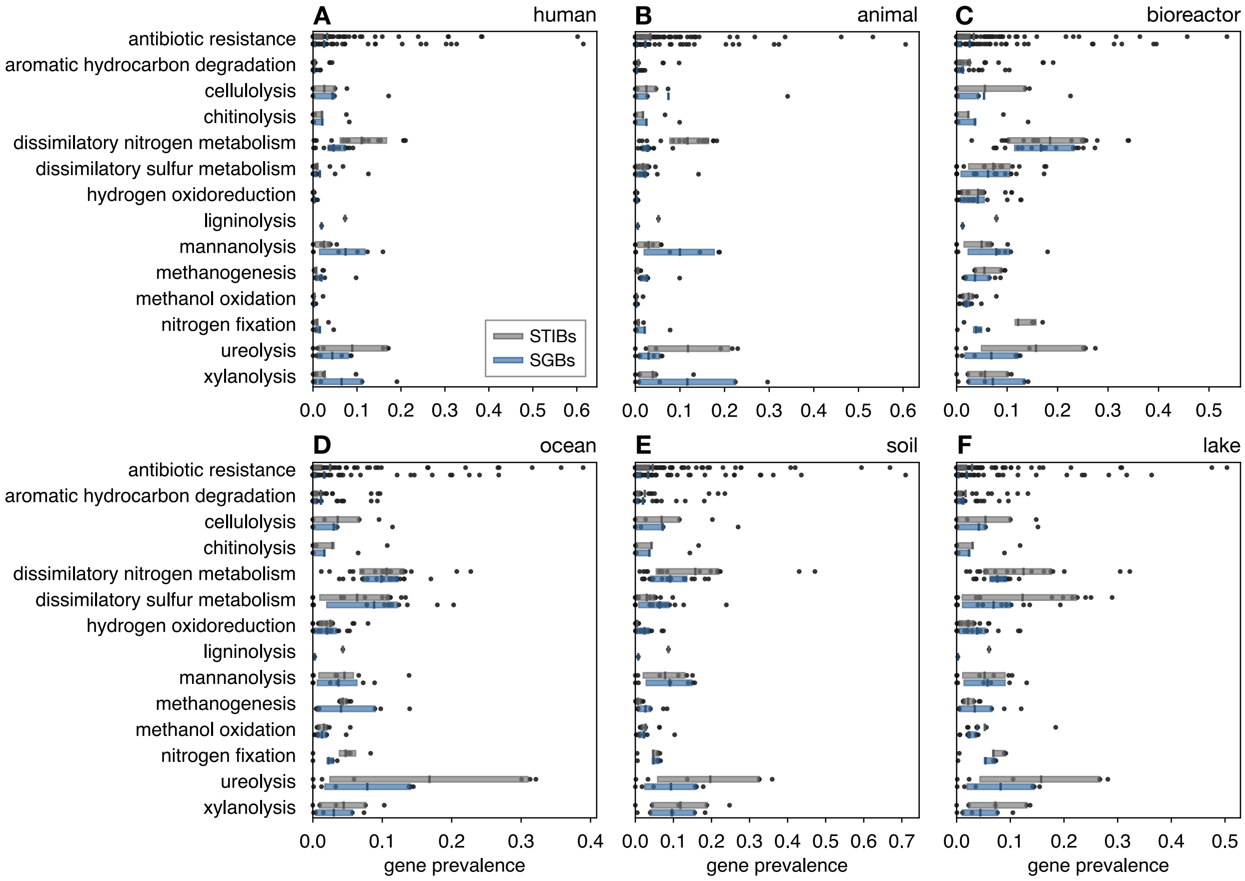

Figure S12: **Gene prevalences for selected metabolic functions (MAG-SGBs vs RefSeq STIBs, size-adjusted).** Box-plots of estimated gene (KEGG ortholog) prevalences based on MAG-SGBs (blue boxes) and RefSeq STIBs (grey boxes, adjusted for the distribution of genome sizes), for selected functions of particular ecological or industrial interest, separately in each environment. Gene prevalences refer to the populations represented by the MAGs and STIBs, i.e., correcting for genome incompleteness. Each box represents a specific function, each point represents a single gene, vertical bar segments denote mean prevalences (i.e., averaged over all genes associated with a specific function), and boxes span the 2nd and 3rd quartile. In most cases gene prevalences are higher among RefSeq STIBs compared to MAG-SGBs, a notable exception being methanogenesis.

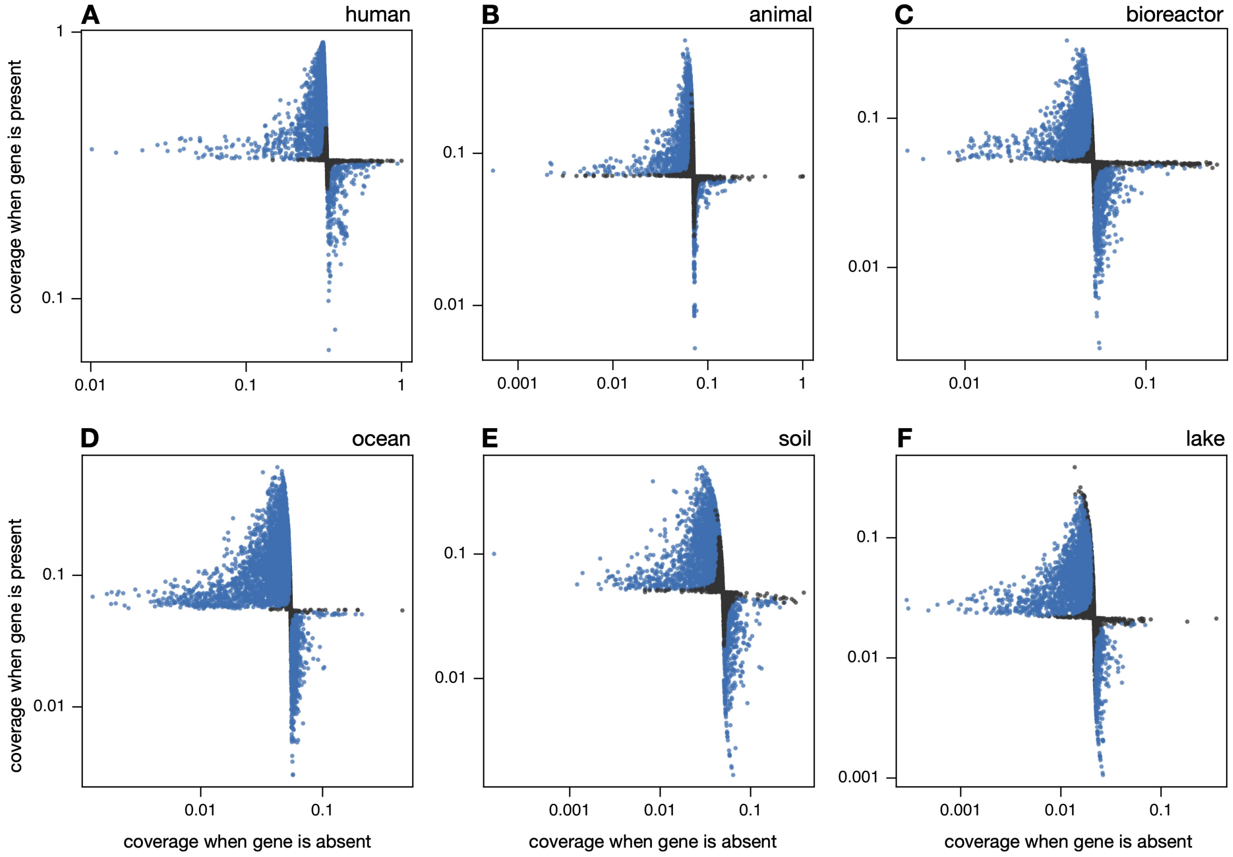

Figure S13: **Gene-dependent coverages.** Estimated probability of a MAG-SGB matching a RefSeq genome at 95% ANI ("coverage") depending on whether a specific gene (KEGG ortholog) is absent (q_0, horizontal axis) or present (q_1, vertical axis) in the complete genome, separately for each environment (one point per gene). Estimates take into account the completeness of MAGs. Note that the center of the "cross" in each figure corresponds to the overall coverage of SGBs regardless of gene presence of absence (q). Note that each gene must either satisfy $q_0 \leq q$ and $q_1 \geq q$, or $q_0 \geq q$ and $q_1 \leq q$, hence the lower-left and upper-right quadrants in the figures are always empty. Blue dots correspond to genes for which q_1 was statistically significantly different from q_0 (under the null model where $q_0 = q_1 = q$), and black dots correspond to genes with no statistically significant difference.

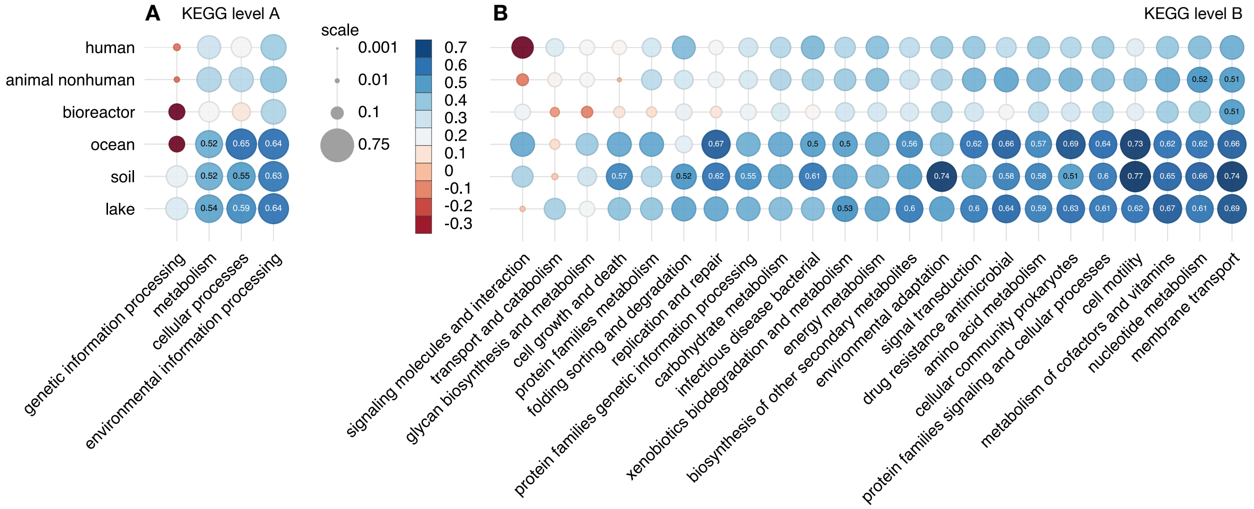

Figure S14: **Median coverage biases by environment and gene category.** Circle chart of median MAG-SGB coverage bias (median β) for each environment and various gene categories of particular interest (defined according to the KEGG hierarchy, levels A and B). Blue colors indicate a positive median β, red colors indicate a negative median β. The size and color saturation of each circle are proportional to the modulus of the median β. Values above 0.5 or below -0.5 are inscribed in the circles. Gene categories are sorted by increasing mean value.

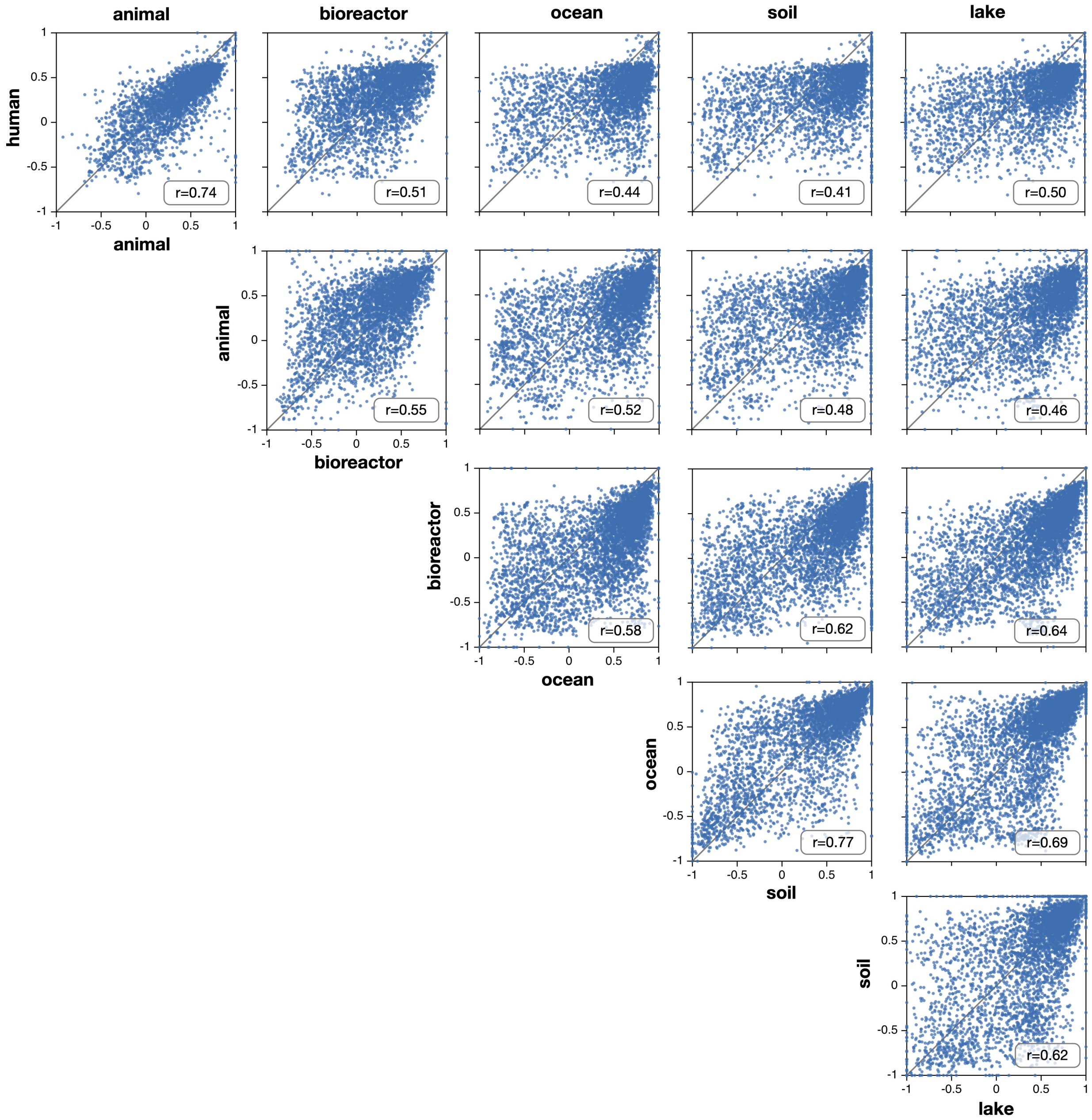

Figure S15: **Gene-specific coverage biases in MAG-SGBs, compared between environments.** Estimated coverage biases (β) compared between environments (one dot per gene, one plot per environment pair). Diagonal lines are shown for reference. The Pearson correlation coefficient (r) between environments is shown in each plot; all correlations were statistically significant ($P < 0.05$) based on a permutation test.

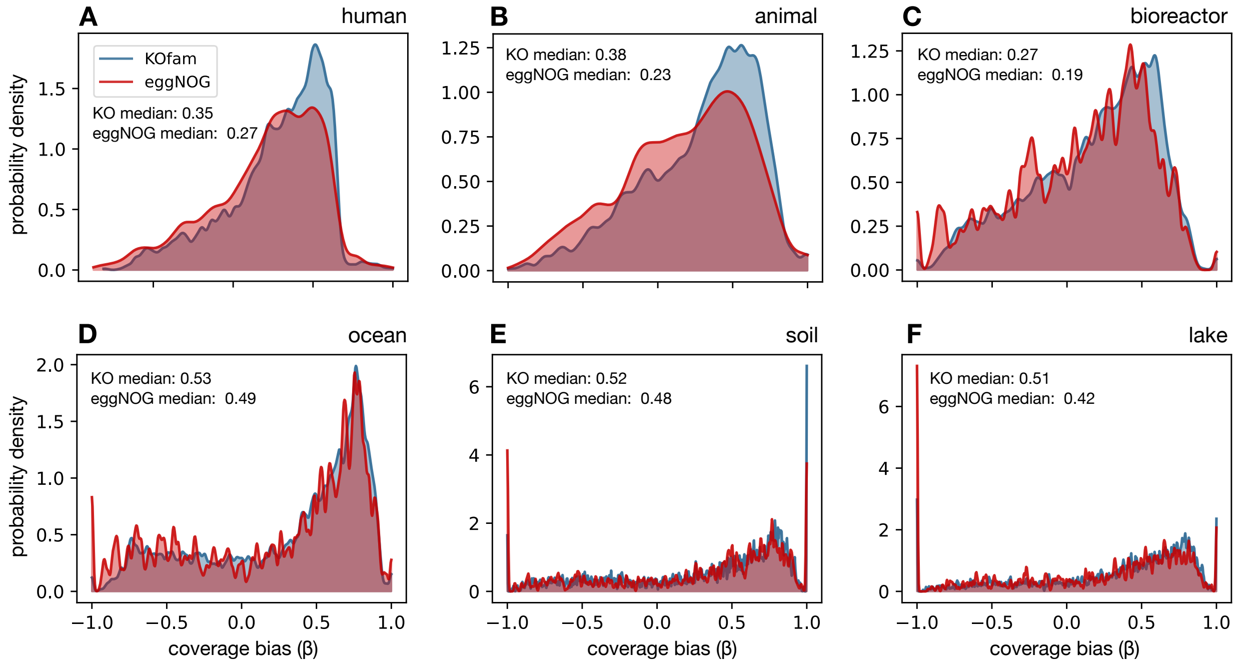

Figure S16: **Distribution of gene-specific coverage biases (KEGG vs eggNOG).** (A) Distribution of gene-specific coverage biases (β) for human-associated MAG-SGBs, i.e., biases in the probability of matching a RefSeq genome at $\geq 95\%$ ANI conditioned on the organism having or lacking a specific gene, considering either KEGG orthologs (as in the main article, blue curve) or alternatively eggNOGs (red curve). For any given gene, a positive bias implies that the probability of an SGB matching a RefSeq genome is greater when the gene is present and smaller when the gene is absent (and vice versa for negative biases). Note that gene presence/absence refers to the population represented by a MAG-SGB, i.e., correcting for MAG incompleteness. (B–F) Similar to (A), but for alternative environments. The median bias β is written in each figure, separately for KEGG orthologs and eggNOGs. Distributions were computed using kernel density estimates over all considered genes. Observe that the coverage biases of most genes are positive, both for KEGG orthologs and eggNOGs.
